# Camptothecin Nanowires Induce the cGAS-STING Pathway to Remold Tumor-Associated Macrophages for Antitumor Immunity

**DOI:** 10.3390/pharmaceutics18060649

**Published:** 2026-05-25

**Authors:** Congyi Zhang, Haotian Wu, Xiaotong Chen, Wenze Yin, Shizhuan Huang, Dixiang Wen, Xueting Song, Xiaoyan Xu, Changmei Zhang, Sheng Tai

**Affiliations:** 1 Department of Hepatopancreatobiliary Surgery, Harbin Medical University Cancer Hospital, Harbin 150081, China; 202001332@hrbmu.edu.cn; 2 Department of Hepatic Surgery, Second Affiliated Hospital of Harbin Medical University, Harbin 150001, China; 2019021266@hrbmu.edu.cn (H.W.); yinwz123456@163.com (W.Y.); hsz0717@163.com (S.H.); wendixiang1998@163.com (D.W.); 3 Department of Children’s and Adolescent Health, Public Health College, Harbin Medical University, Harbin 150086, China; cxthmu@163.com (X.C.); songxueting0105@163.com (X.S.); 4 Department of Pharmaceutics, Harbin Medical University-Daqing Campus, 1 Xinyang Rd, Daqing 163319, China; xxykadw95@163.com

**Keywords:** camptothecin nanowires, sialic acid, tumor-associated macrophages, cGAS-STING signaling pathway, immune escape

## Abstract

**Background/Objectives**: This study aimed to develop a novel tumor-associated macrophage (TAM)-targeting nanoplatform to improve the solubility and bioavailability of camptothecin (CPT) and achieve active targeted drug delivery for enhanced anti-tumor immunotherapy. **Methods**: We constructed a sialic acid-disulfide bond-camptothecin (SA-SS-CPT) nanowire system. Sialic acid was used as a targeting ligand to specifically recognize the overexpressed Siglec-E receptor on TAMs. Upon cellular internalization, the disulfide bond was designed to respond to intracellular glutathione (GSH), enabling controlled drug release. **Results**: The SA-SS-CPT nanowires significantly improved CPT solubility and enabled targeted delivery to TAMs. Following GSH-responsive cleavage and CPT release, the nanowires induced DNA damage in TAMs, activating the cGAS-STING signaling pathway. This promoted TAM polarization toward the M1 phenotype, enhanced pro-inflammatory and anti-tumor immune responses, and inhibited tumor immune escape. Furthermore, SA-SS-CPT synergistically improved the efficacy of PD-L1 blockade immunotherapy, remodeling the tumor immune microenvironment. **Conclusions**: The SA-SS-CPT nanoplatform effectively targets TAMs, repolarizes them to an anti-tumor M1 phenotype, and activates the cGAS-STING pathway. It shows strong potential for overcoming tumor immune escape and synergizing with PD-L1 checkpoint blockade to achieve significant tumor clearance.

## 1. Introduction

Liver cancer is the fifth most common cancer and the second leading cause of cancer-related deaths in Asia. Data from 2020 show that 72.5% of global liver cancer cases are concentrated in Asia, with hepatocellular carcinoma (HCC) being the predominant pathological type [1]. Currently, treatment options for advanced HCC are limited, highlighting an urgent need to develop new drugs and therapeutic strategies. Although targeted therapy and immunotherapy have shown potential, further exploration is required to determine their optimal application and therapeutic targets [2].

In recent years, immunotherapy, which aims to restore the immune system’s ability to recognize and kill tumor cells, has played a significant role in the treatment of HCC. Among immune components, macrophages are crucial to immunity and are ubiquitous in peripheral tissues, serving as the first line of defense against pathogens. In the tumor microenvironment, tumor-associated macrophages (TAMs) are categorized into two main functional phenotypes: M1 and M2. M1 macrophages are generally considered to have anti-tumor effects [3,4], whereas M2 macrophages tend to promote tumor growth and metastasis [5,6]. In the tumor microenvironment, TAMs are often polarized toward the M2 phenotype, which exhibits tumor-promoting properties [7]. Therefore, reprogramming TAMs from M2 polarization into the tumor-suppressive M1 phenotype represents an important approach in cancer therapy.

Sialic acid (SA) is a derivative of neuraminic acid and a type of nine-carbon monosaccharide widely found at the terminal structures of glycoproteins and glycolipids. It plays important roles in various physiological and pathological processes [8]. In recent years, the role of SA in cancer has garnered significant attention, particularly in HCC, where abnormal SA expression has been closely associated with cancer progression, invasiveness, and prognosis [9]. Studies have revealed that tumor cells, through surface-expressed SA, interact with Siglec receptors on macrophages, further promoting M2 polarization. This process suppresses the immune system’s attack on tumor cells, facilitating immune escape. Therefore, targeting the SA-Siglec interaction may offer a strategy to reprogram TAMs towards the M1 phenotype, potentially improving the tumor immune microenvironment [10].

Siglec receptors are primarily expressed on the surface of immune cells such as macrophages, dendritic cells, and natural killer cells [11]. The extracellular domain of Siglec receptors can specifically recognize sialic acid (SA), while their intracellular domain typically contains an immunoreceptor tyrosine-based inhibitory motif, which mediates the negative regulation of immune responses [12]. Through inhibitory signal transduction, Siglec receptors play a crucial role in maintaining immune homeostasis and preventing excessive inflammatory reactions [11]. This mechanism is similar to that of immune checkpoints, making Siglec a part of the immune escape process. In HCC, high SA expression activates Siglec-10, inhibiting the phagocytic activity and pro-inflammatory responses of TAMs, thereby promoting tumor immune escape [13]. The interaction between Siglec-E and tumor-associated dendritic cells suppresses antigen presentation, further impairing the immune system’s ability to recognize HCC [14]. Numerous studies have highlighted the potential of targeting the SA-Siglec axis to modulate macrophage immune function, which may provide new directions for the development of novel cancer immunotherapies [15].

Currently, therapeutic agents for HCC include camptothecin (CPT). CPT is a topoisomerase I inhibitor that exerts its anti-tumor effects by interfering with DNA replication processes [16,17]. Additionally, studies such as that by Chen [18] have reported that CPT influences macrophage function by inhibiting nitric oxide biosynthesis in RAW 264.7 macrophages. However, whether CPT participates in anti-tumor activity by modulating the polarization pathway of tumor-associated macrophages remains unreported. Although CPT has demonstrated potent anti-tumor activity in cancer treatment, its direct application in HCC therapy faces several challenges. First, CPT exhibits extremely poor water solubility, leading to low absorption and bioavailability in vivo, which limits its broad clinical use. Furthermore, CPT has high toxicity, particularly toward normal cells, significantly restricting its therapeutic applicability [19]. Therefore, researchers are actively exploring various strategies to enhance the safety and efficacy of CPT in cancer treatment.

**Scheme 1:**
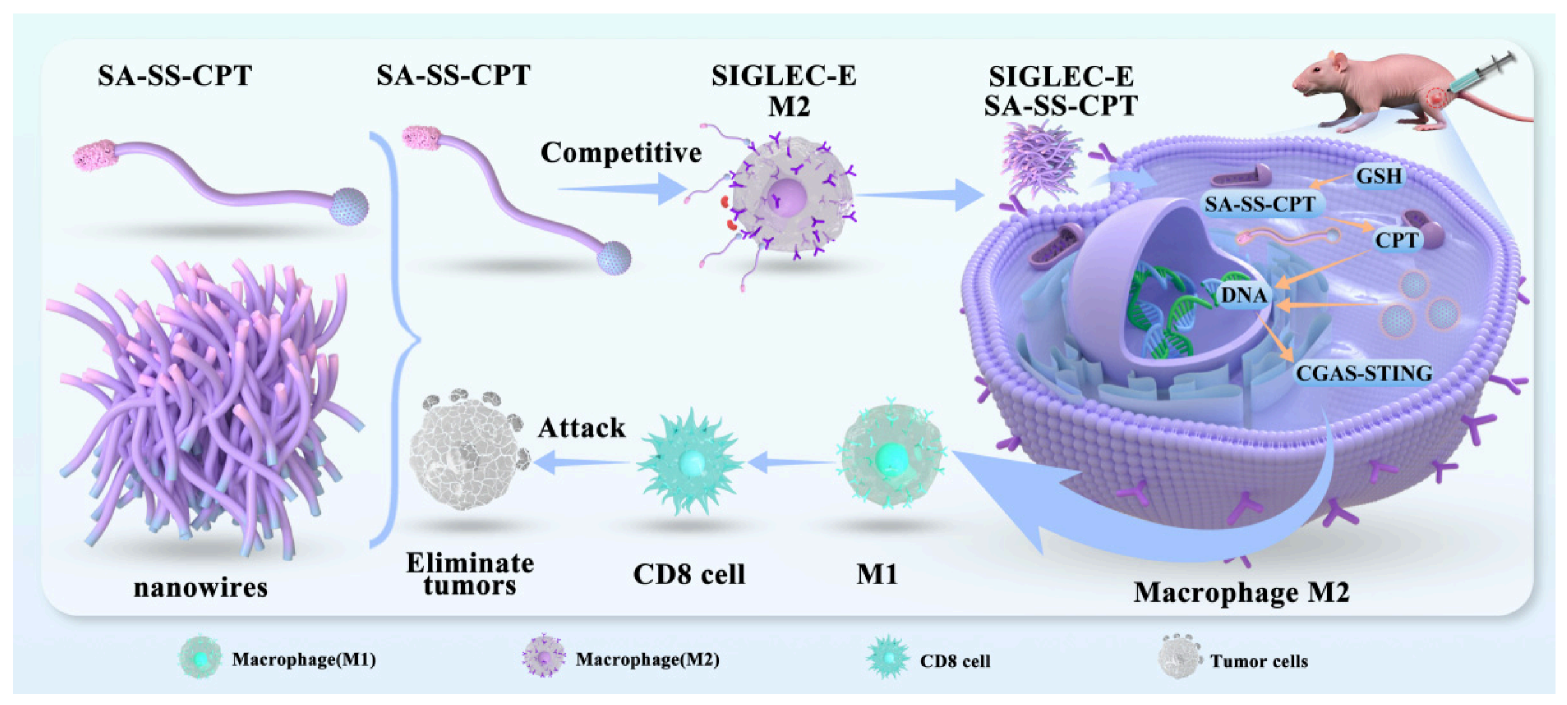
Mechanism of camptothecin nanowires remodeling tumor-associated macrophage polarization via the cGAS-STING signaling pathway to promote anti-tumor immunity.

Nanomedicine-based drug delivery systems have emerged as a pivotal area in pharmaceutical research in recent years and are frequently employed to overcome the limitations of active natural products. This study aims to design nanoparticles with active targeting capability toward TAMs to address the challenges of CPT, including its high toxicity and poor water solubility. A chemical synthesis approach was used to conjugate CPT with SA via a disulfide bond, resulting in SA-SS-CPT. The SA modification enables the nanoparticles to competitively target macrophages over tumor cells, reducing the binding opportunity between tumor-derived SA and Siglec receptors on TAMs, thereby inhibiting tumor immune evasion. The disulfide bond (-SS-) in SA-SS-CPT is cleaved by intracellular glutathione (GSH) within TAMs, leading to the release of CPT [20,21]. SA-SS-CPT induces DNA damage in TAMs, subsequently activating the cGAS-STING signaling pathway. This promotes the repolarization of TAMs toward the M1 phenotype, enhancing their pro-inflammatory response and anti-tumor immune activity, which effectively counteracts immune escape. Furthermore, SA-SS-CPT nanoparticles synergize with PD-L1 blockade immunotherapy to jointly remodel the tumor immune microenvironment, ultimately leading to significant tumor clearance, as illustrated in Scheme 1.

However, we hypothesized that presenting sialic acid on the surface of nanowires could convert this immunosuppressive interaction into an active targeting and immunomodulatory signal. By competitively occupying Siglec-E receptors, SA-SS-CPT not only blocks the inhibitory crosstalk originating from tumor-derived SA but also triggers receptor-mediated internalization, allowing subsequent drug release and macrophage repolarization. Unlike existing CPT prodrugs that generally rely on PEGylation or passive targeting, this work utilizes SA as an active targeting ligand, which not only achieves specific recognition of TAMs but also directly intervenes in immunosuppressive signaling pathways by competitively inhibiting the interaction between tumor-derived SA and Siglec-E—an immunological modulation dimension not addressed by other CPT prodrug designs. Furthermore, while most CPT prodrugs depend on nanocarriers (such as silica, liposomes, or micelles) for encapsulation, SA-SS-CPT exists as a carrier-free self-assembling system, which simplifies the formulation process and reduces carrier-associated toxicity.

## 2. Materials and Methods

### 2.1. Materials

(S)-(+)-Camptothecin (Aladdin, Shanghai, China), N-Acetylneuraminic acid (Aladdin, Shanghai, China), Anti-PD-1 (catalog no. BE0146, Bio X Cell, Lebanon, NH, USA), HiScript III RT SuperMix for qPCR (+gDNA wiper (R323-01), ChamQ Universal SYBR qPCR Master Mix (Q711-02) (Vazyme, Nangjing, China), 3,3′-Dioctadecyloxacarbocyanine perchlorate (DiO) (Sigma-aldrich, St. Louis, MO, USA), 1,1′-Dioctadecyl-3,3,3′,3′-tetramethylindotricarbocyanine iodide (DiR) (Sigma-aldrich), Cell Counting Kit-8 assay (Sigma-aldrich, St. Louis, MO, USA), TNF-α, IL-1β, IL-1RA, CD33, CD169, and β-Tubulin primary antibody (HUABIO, Hangzhou, China).

### 2.2. Animals

Twenty BALB/c nude mice, male, 20–30 g, 4 weeks of age, C57BL/6J (C57) mice, male, 20–30 g, 4 weeks of age, SPF grade, were purchased from Beijing Weitonglihua Laboratory Animal Technology Co., Ltd. (China, Beijing). All mice were kept in the SPF animal room with a 12 h light/dark cycle, maintained at a temperature range of 18–22 °C and humidity levels of 50–60%. The animal experiment was approved by the Animal Ethics Committee of the Second Affiliated Hospital of Harbin Medical University (YJSKY2024-433). All procedures involving anesthesia and euthanasia were conducted in strict accordance with the American Veterinary Medical Association (AVMA) Guidelines for the Euthanasia of Animals. For surgical anesthesia, mice were administered sodium pentobarbital (40–80 mg/kg) via intraperitoneal injection, and the depth of anesthesia was assessed by the absence of pedal withdrawal reflex. At the experimental endpoints, euthanasia was humanely performed by an overdose of the same anesthetic at a dose exceeding 150 mg/kg. Death was confirmed by the absence of pupillary light reflex and cessation of heartbeat.

### 2.3. Methods

#### 2.3.1. Synthesis of Camptothecin Nanowires

The synthetic route to Sialic Acid-SS-Camptothecin (SA-SS-CPT) is shown in Scheme S1. Sialic acid (2 g, 6.5 mmol) was dissolved in methanol (30 mL) under an argon atmosphere. Trifluoroacetic acid (0.1 mL) was added dropwise at room temperature, and the reaction was stirred overnight at the same temperature. After completion of the reaction, the solvent was removed under reduced pressure, and the crude product was used directly in the next step.

The resulting compound **1** was dissolved in pyridine (20 mL), followed by the addition of acetic anhydride (6 mL, 65 mmol) and DMAP (800 mg, 6.5 mmol). The mixture was maintained under argon and stirred overnight at room temperature. Methanol was added to quench the reaction, and the solvent was evaporated under reduced pressure at 70 °C. The crude product was purified by silica gel column chromatography using a gradient of ethyl acetate in petroleum ether (10% to 100%) to afford compound **2** as a white solid (1.6 g, 46.4% yield over two steps).

Compound **2** (1.6 g, 3 mmol) was dissolved in dichloromethane (30 mL), and butynol (420 mg, 6 mmol) was added. The solution was cooled to 0 °C under argon, and boron trifluoride diethyl etherate was added dropwise. The reaction was then warmed to room temperature and stirred overnight. Upon completion, the mixture was poured into ice water and basified to alkaline pH using saturated sodium bicarbonate solution. The aqueous layer was extracted twice with dichloromethane, and the combined organic extracts were concentrated under reduced pressure. Purification by silica gel column chromatography (ethyl acetate/petroleum ether, 10% to 100%) gave compound **3** as a white solid (1.2 g, 73.6% yield).

Compound **3** (1.2 g, 2.2 mmol) was dissolved in methanol (30 mL), and sodium methoxide (118 mg, 1 mmol) was added. The reaction was stirred at room temperature overnight. Methanol was then removed under reduced pressure, and the residue was carried forward without further purification.

The resulting compound **4** was dissolved in water (10 mL), and 4 M sodium hydroxide solution (1 mL) was added. After stirring at room temperature overnight, the reaction was quenched by adding cation exchange resin until the mixture reached neutral pH. The mixture was filtered, and the filtrate was concentrated to give compound 5 (600 mg, 75.2% yield over two steps).

Compound **5** (120 mg, 0.33 mmol) was dissolved in water (3 mL), followed by the sequential addition of N,N-dimethylformamide (6 mL), compound **6** (200 mg, 0.33 mmol), 0.3 M anhydrous copper (II) sulfate (334 μL, 0.1 mmol), and sodium ascorbate (65 mg, 0.33 mmol). The reaction was allowed to proceed at 40 °C overnight. After removal of the solvent under reduced pressure, the residue was dissolved in N,N-dimethylformamide (5 mL) and purified by reverse-phase C18 medium-pressure flash chromatography using a gradient of methanol in water (5% to 95%). The purified product was lyophilized to afford the final compound 7 (SA-SS-CPT) as a solid. The purity of all intermediate compounds and the final product was confirmed by proton nuclear magnetic resonance spectroscopy (^1^H-NMR, 400 MHz) and MS-HPLC analysis. The ^1^H-NMR measurements were performed on a Bruker 400 MHz AVANCE III spectrometer (Bruker Corporation, Billerica, MA, USA) under the following conditions: approximately 10 mg of sample was placed into an NMR tube, dissolved in 0.5 mL of deuterated dimethyl sulfoxide (DMSO-d_6_) with vortexing to ensure complete dissolution. The ^1^H resonance frequency was 400 MHz, with 16 scans and a relaxation delay of 2 s. MS-HPLC analysis was conducted on a Waters LC-MS system equipped with a Waters Acquity™ UPLC BEH C18 column (Waters Corporation, Milford, MA, USA). The procedure was as follows: precisely 5 mg of sample was fully dissolved in 10 mL of methanol, and 200 µL of the resulting solution was transferred into a micro-insert placed in an HPLC vial. The injection volume was 5 µL.

#### 2.3.2. FTIR and UV Characterization

Dried SA-SS-CPT was thoroughly ground with spectrally pure potassium bromide (KBr) powder at a mass ratio of approximately 1:100 (sample/KBr, *w*/*w*). The mixture was then compressed into transparent pellets under a hydraulic press at 8–10 tons of pressure for 2 min. Fourier transform infrared (FTIR) spectra were recorded using a Nicolet iS50 FTIR spectrometer (Thermo Fisher Scientific, Waltham, MA, USA). Spectra were acquired in transmission mode over a wavenumber range of 4000–400 cm^−1^ at a resolution of 4 cm^−1^, with 32 scans co-added per spectrum. The background spectrum (pure KBr pellet) was subtracted automatically. SA-SS-CPT nanowires (1 mg/mL, 1 mL) were dissolved in a suitable amount of methanol, and full-wavelength scanning was performed using a UV-visible spectrophotometer (scan range: 200–600 nm).

#### 2.3.3. Preparation and Characterization of SA-SS-CPT Nanowires

SA-SS-CPT nanowires were prepared by dissolving 5 mg of SA-SS-CPT in 1 mL of ultrapure water, followed by probe sonication on ice (using a Branson SFX250 probe sonicator (Branson Ultrasonics Corporation, Danbury, CT, USA) with a 3 mm diameter tip; amplitude: 30%; pulse cycle: 5 s on/5 s off for a total of 30 cycles; total sonication time: 5 min). For DLS and zeta potential measurements, the nanowire dispersion was diluted 10-fold with ultrapure water (refractive index: 1.330, viscosity: 0.8872 cP at 25 °C). The hydrodynamic diameter and polydispersity index (PDI) were determined using a Marvin nanoparticle size analyzer (Malvern Panalytical, Malvern, UK) at a backscatter angle of 173°. The refractive index of the SA-SS-CPT material was set to 1.48, and the absorption was set to 0.01. Zeta potential was calculated using the Smoluchowski model (F(ka) = 1.5). All measurements were performed in triplicate at 25 °C.

The morphology of the SA-SS-CPT nanowires was observed under a tungsten-filament transmission electron microscope (JEM1200EX, JEOL, Tokyo, Japan) after negative staining. A total of 5 µL of sample was placed on a carbon-coated copper grid for 2 min, excess liquid was blotted, followed by staining with 5 µL of 1% (*w*/*v*) uranyl acetate for 1 min, then air-dried. To evaluate the stability of the SA-SS-CPT nanowires, their average size and zeta potential were measured at days 0, 3, 6, 9, 12, 15, 18, 21, 24, 27, and 30 during storage (at 4 °C in the dark).

#### 2.3.4. Hemolysis Assay

A hemolysis assay was performed to evaluate the biocompatibility of the nanowires. Briefly, mouse blood was collected via cardiac puncture into heparinized tubes. A 2% erythrocyte suspension was prepared by centrifuging the blood (1000× *g*, 10 min, 4 °C), washing the erythrocytes with normal saline, and resuspending them. This suspension was incubated with various concentrations of SA-SS-CPT nanowires (0, 10, 50, 100, 200, 500 µg/mL) at 37 °C for 1 h (in a water bath with gentle shaking every 10 min). The samples were then centrifuged (1000× *g*, 5 min), and the absorbance of the supernatants was measured at 540 nm by SYNERGY microplate reader (BIOTEK, Winooski, VT, USA). Distilled water and normal saline served as the positive and negative controls, respectively. The hemolysis rate (%) = (A_sample_ − A_negative_)/(A_positive_ − A_negative_) × 100%.

#### 2.3.5. Camptothecin Release

Standard CPT solutions at varying concentrations (0, 1, 2, 5, and 10 μg/mL) were prepared separately. The absorbance of these solutions was measured at a wavelength of 257 nm using a UV-Vis spectrophotometer to establish a standard curve. Subsequently, 1 mg/mL of the SA-SS-CPT solution was placed in a centrifuge tube, to which a 10 mM GSH solution was added. After thorough mixing, the tube was incubated in a constant temperature shaker at 37 °C. The supernatant (100 μL) was sampled at specific time intervals (0, 15, 30, 45, 90, and 135 min) after the reaction initiation. The absorbance of the reaction solution at 257 nm was measured for each sample by SYNERGY microplate reader (BIOTEK, USA). The concentration of released CPT was calculated based on the linear regression equation derived from the standard curve.

#### 2.3.6. Pharmacokinetics Study

BALB/c nude mice were intravenously administered SA-SS-CPT via the tail vein. Blood samples were collected at predetermined time points (0, 1, 2, 3, 6, 8, 12, and 24 h) post-injection. Plasma was separated from the blood samples, treated with methanol, and centrifuged. The resulting supernatant (20 µL injection volume) was analyzed using High-performance liquid chromatography (HPLC system: Agilent 1260 Infinity II; column: Zorbax Eclipse Plus C18 (4.6 mm × 150 mm, 3.5 µm); mobile phase: acetonitrile/water (40:60 *v*/*v*) with 0.1% formic acid; flow rate: 1.0 mL/min; column temperature: 30 °C; detection wavelength: 257 nm; run time: 10 min).

#### 2.3.7. Immunofluorescence Analysis of Siglec-E Expression

A co-culture system was established using RAW 264.7 cells (1 × 10^5^ cells/mL) and Hepa1–6 cells (5 × 10^4^ cells/mL), with the latter seeded in the upper chamber of a 6.5 mm diameter, 8 µm pore size transwell insert, for 24 h under standard conditions (37 °C, 5% CO_2_). Following co-culture, the RAW 264.7 cells on poly-L-lysine-coated coverslips were fixed with 4% paraformaldehyde, permeabilized with 0.1% Triton X-100 (10 min), and blocked with 5% BSA (1 h at room temperature). The cells were subsequently incubated with an anti-Siglec-E primary antibody (1:200 dilution in 1% BSA) at 4 °C overnight. After PBS washes, a fluorescent secondary antibody was applied in the dark. Imaging was performed using a Confocal laser scanning microscope (DeltaVision Ultra, Cytiva, Marlborough, MA, USA).

#### 2.3.8. Cellular Uptake of SA-SS-CPT

RAW 264.7 cells in the logarithmic growth phase were seeded into 12-well plates at a density of 1 × 10^5^ cells/mL and cultured for 24 h in a 5% CO_2_ incubator at 37 °C. Subsequently, the cells were treated with 20 µL of DiO-labeled SA-SS-CPT nanowires (prepared by mixing 1 mg/mL DiO with SA-SS-CPT at 1:10 ratio during nanowire formation, followed by dialysis to remove free DiO) and further incubated for 4 h at 37 °C. After incubation, the culture medium was removed, and the cells were washed twice with PBS, trypsinized, and centrifuged. The resulting cell pellet was resuspended in 200 μL of PBS. Cellular uptake was analyzed using a Flow cytometry (Beckman Coulter, Brea, CA, USA) (excitation 488 nm, emission 530 nm). 

#### 2.3.9. In Vivo Imaging

Hepa1–6 cells (5 × 10^6^ cells in 100 µL PBS) were subcutaneously inoculated into BALB/c nude mice at five weeks of age. Seven weeks post-inoculation, once the tumor model was successfully established, the model mice were intravenously administered SA-SS-CPT@DiR solutions. Briefly, the SA-SS-CPT@DiR complex was prepared by mixing DiR solution (1 mg/mL) with SA-SS-CPT solution (1 mg/mL) in chloroform at a 10:1 volume ratio. After chloroform evaporation, the resulting film was reconstituted in 1 mL of physiological saline. Free DiR was removed by centrifugation at 2800× *g* for 5 min. The formulated solution was injected via the tail vein at a DiR dose of 20 mg/kg body weight. Fluorescence imaging was performed using a Carestream In Vivo FX Professional Imaging System (Bruker Corporation, Billerica, MA, USA) with excitation and emission wavelengths set at 720 nm and 790 nm, respectively. Mice were anesthetized with 2% isoflurane during imaging, and images were acquired at 1, 4, 8, 12, 24 and 36 h post-injection.

#### 2.3.10. In Vitro Cytotoxicity of SA-SS-CPT

The in vitro cytotoxicity of SA-SS-CPT nanowires was determined by a CCK-8 assay. HCCLM3 cells, plated in 96-well plates (1 × 10^4^ cells/mL) for 24 h, were treated with a series of nanowires concentrations (1920–15 μg/mL) for an additional 24 h. Following the treatment, RAW 264.7 cells were incubated with CCK-8 reagent for 1 h at 37 °C. The absorbance was measured at 450 nm. Cell viability was expressed as a percentage relative to untreated controls, calculated using the formula: Viability (%) = [(A_drug_ − A_blank_)/(A_control_ − A_blank_)] × 100%.

#### 2.3.11. 3D Nanolive Imaging for Macrophage Polarization

RAW 264.7 cells were adjusted to a density of 5 × 10^4^ cells/mL in DMEM with 10% FBS and plated in a Nanolive-specific dish. Following a 24 h adhesion period (37 °C, 5% CO_2_), the cells were stimulated with 30 μg/mL SA-SS-CPT for 24 h. Morphological changes and cell state were subsequently visualized and analyzed with a Nanolive 3D Cell Explorer microscope (Nanolive SA, Lausanne, Switzerland).

#### 2.3.12. RNA-Seq Analysis

RAW 264.7 cells were either untreated (Control) or treated with SA-SS-CPT for 24 h (Drug). Cell pellets, stored at −80 °C, were sent for RNA sequencing (Shanghai Biochip Co. Shanghai, China). DEGs were called using DESeq2 (R package, accessed on 31 October 2025) with criteria of |log2FC| ≥ 1 and *p* < 0.05. GO enrichment analysis for the DEGs was performed using a hypergeometric test to identify significantly enriched terms relative to the genomic background.

#### 2.3.13. Immunofluorescence Analysis

After a 24 h intervention of RAW 264.7 cells with SA-SS-CPT (7.5, 15, and 30 μg/mL), the cells on coverslips were processed for immunofluorescence. Briefly, cells were fixed with 4% PFA, permeabilized with 0.1% Triton X-100, and blocked with 5% BSA. Staining was performed using a primary antibody against γ-H2AX (overnight, 4 °C) followed by a fluorescent secondary antibody (room temperature, in the dark). Imaging was conducted with a Confocal laser scanning microscope (DeltaVision Ultra, GE, USA).

#### 2.3.14. Detection of DNA Damage and the cGAS-STING Pathway

After reaching an appropriate confluence, RAW 264.7 cells were treated with SA-SS-CPT or CPT at concentrations of 7.5, 15, and 30 μg/mL for 24 h. Subsequently, total protein was extracted from the cells for subsequent analysis. Equal amounts of proteins (40 μg/well) and protein marker were taken for SDS-PAGE electrophoresis and then transferred to a PVDF membrane. The membrane was blocked with 5% BSA at room temperature for 1 h, and then incubated overnight on a shaker at 4 °C with γ-H2AX, IRF-3, TBK-1, P-STING, STING, CD206, CD86, TNF-α, IL-1β monoclonal primary antibody (1:1000). After thorough washing with TBST, the membrane was incubated with secondary antibody (1:50,000) at room temperature in the dark for 1 h. The ECL chemiluminescence method was used for color development. The Image J software 5.2.1 was used to analyze the gray value of the target band, and β-tubulin was used as an internal reference for standardized quantification.

#### 2.3.15. RT-PCR Analysis of Inflammatory Factors

Total mRNA from SA-SS-CPT-treated RAW 264.7 cells was isolated with TRIzol. After reverse transcription (PrimeScript™ Kit; 37 °C, 15 min), qPCR was performed using gene-specific primers (Table 1) under standard cycling conditions (95 °C for 10 min; 40 cycles of 95 °C/15 s and 60 °C/60 s). Gene expression (Arg-1, IL-1ra, CD163, iNOS, TNF-α, IL-6) was normalized to β-actin and calculated via the 2^−ΔΔCT^ method.

#### 2.3.16. Establishment of Tumor-Bearing Mouse Model

A tumor-bearing mouse model was established by subcutaneously injecting Hepa1–6 cells (1 × 10^6^) into the right posterior flank of BALB/c nude mice, which was designated as day 0. Seven days post-inoculation, once the model was successfully established, the mice were randomly divided into three groups (*n* = 6): Con, CPT, and SA-SS-CPT. Treatments were administered via tail vein injection at a dose of 20 mg/kg. The administration continued until day 22, after which subsequent experiments were performed. Tumor volume was monitored and calculated using the formula: V = L × W^2^/2, where V represents the tumor volume, L is the longest diameter, and W is the shortest diameter. Additionally, tumor growth was monitored by bioluminescent imaging using an in vivo imaging system.

#### 2.3.17. Hematoxylin and Eosin (H&E) Staining

Paraffin sections of 5 μm thickness were deparaffinized in graded ethanol (100%, 95%, 75%), stained with hematoxylin, rinsed, and dehydrated through an ascending ethanol series (75% to absolute). After clearing in xylene, sections were mounted with neutral balsam for imaging.

#### 2.3.18. Ki67 Immunohistochemical Staining

Paraffin sections were deparaffinized in xylene and rehydrated. Frozen sections were air-dried and fixed with 4% PFA. All sections underwent antigen retrieval, endogenous peroxidase blockade (3% H_2_O_2_), and serum blocking. Incubation with anti-Ki67 primary antibody (4 °C overnight) was followed by an HRP-conjugated secondary antibody and DAB development for microscopic examination.

#### 2.3.19. Immunohistochemistry for CD86

Following deparaffinization in xylene and rehydration through an ethanol gradient, tissue sections were subjected to antigen retrieval. Endogenous peroxidases were blocked with 3% H_2_O_2_ (10–15 min, RT). After PBS washing, sections were blocked with 5%BSA and then incubated with an anti-CD86 primary antibody overnight at 4 °C. After PBS washes, a secondary antibody was applied 60 min. Signal was visualized using DAB, and sections were examined microscopically.

#### 2.3.20. Analysis of Liver Metastasis

A liver metastasis model was established by intrasplenic injection of 1 × 10^6^ Hepa1–6 cells into BALB/c-nu/nu nude mice (day 0). Pharmacological intervention began on day 4 post-inoculation. The animals were randomly assigned to three groups: a Control group administered normal saline, a CPT group (5 mg/mL), and an SA-SS-CPT group (5 mg/mL). All formulations were delivered intravenously at a dosage of 20 mg/kg every other day for a 12-day period. Tumor progression was assessed non-invasively via fluorescence imaging. On day 16, mice were euthanized under pentobarbital anesthesia. Livers were excised to evaluate metastatic nodule formation, liver weight, and maximum tumor size. Body weights were recorded throughout the study. Liver sections were prepared for histopathological examination using H&E staining and for assessment of macrophage polarization via CD86 immunohistochemical staining.

#### 2.3.21. SA-SS-CPT Enhances the Immunotherapeutic Efficacy of PD-L1 in Mice

A hepatocellular carcinoma model was established in male C57BL/6 mice by hydrodynamic tail vein injection of plasmid DNA (dissolved in sterile physiological saline). The plasmids (Sangon Biotech, Shanghai, China) included C-Myc (18 μg), sgP53 (18 μg), and SB13 (7.5 μg) per mouse, delivered in a volume equivalent to 10% of the body weight within 6–7 s. Starting on day 7, mice were administered PD-L1, SA-SS-CPT, or PD-L1 & SA-SS-CPT until day 22. On day 24, in vivo imaging was conducted, after which liver tissues were collected for analysis of liver weight, maximum tumor size, and CD8^+^ immunohistochemistry.

### 2.4. Statistical Analysis

Data from three independent replicates are expressed as mean ± SD. GraphPad Prism 7 and SPSS 21.0 were used for statistical analysis. Inter-group comparisons were made by Student’s *t*-test or one-way ANOVA, as appropriate. Repeated-measures ANOVA with Bonferroni correction was used for multi-timepoint comparisons, and Spearman’s test was used for correlation analysis. A *p*-value < 0.05 was defined as statistically significant.

## 3. Results

### 3.1. Synthesis of SA-SS-CPT

**Figure 1:**
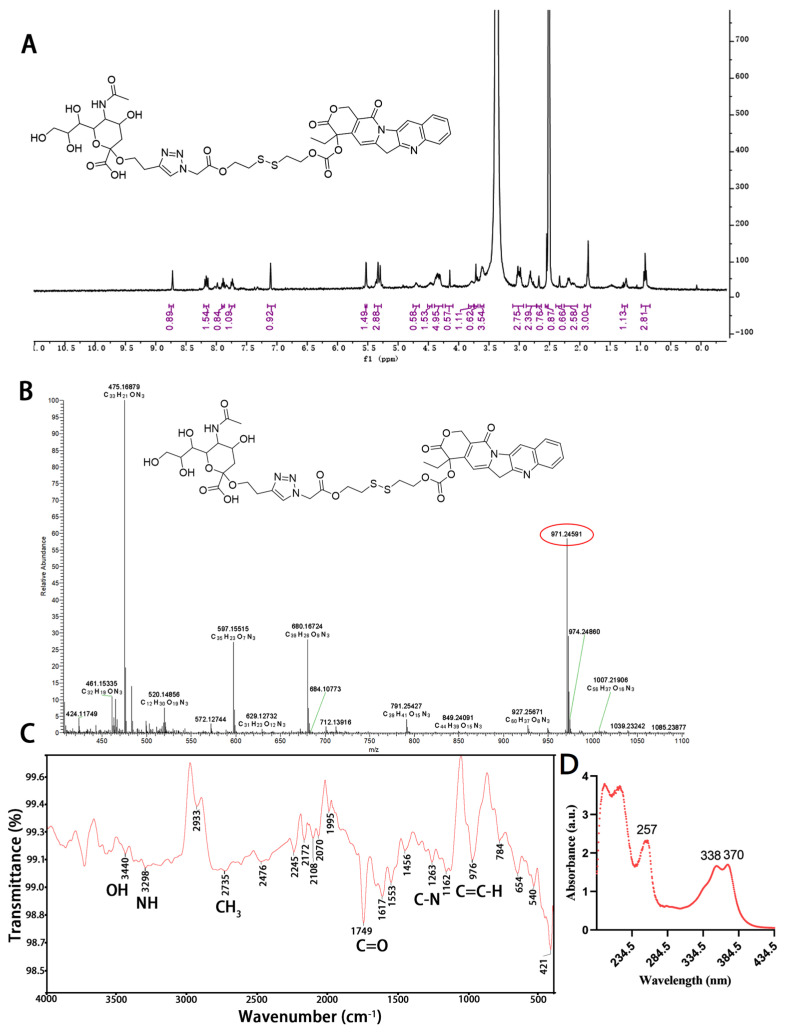
(**A**) ^1^H NMR (400 MHz, DMSO-d6) of SA−SS−CPT. (**B**) MS spectra of SA−SS−CPT. (**C**) Infrared spectroscopy of SA−SS−CPT. (**D**) Ultraviolet absorption spectrum of SA−SS−CPT.

The compound SA-SS-CPT was obtained as a pale yellow solid (110 mg, 34.4% yield). ^1^H NMR (400 MHz, DMSO-d6) δ 8.72 (s, 1H), 8.21–8.12 (m, 2H), 7.89 (t, J = 7.6 Hz, 1H), 7.74 (t, J = 7.6 Hz, 1H), 7.10 (s, 1H), 5.53 (s, 1H), 5.31 (d, J = 14.1 Hz, 3H), 4.70 (s, 1H), 4.46 (s, 2H), 4.34 (td, J = 9.7, 3.5 Hz, 5H), 4.15 (s, 1H), 3.79 (s, 1H), 3.70 (d, J = 10.2 Hz, 1H), 3.61 (s, 4H), 3.11–2.93 (m, 3H), 2.89–2.72 (m, 2H), 2.68 (s, 1H), 2.55 (s, 1H), 2.33 (s, 1H), 2.26–2.04 (m, 3H), 1.87 (d, J = 2.5 Hz, 3H), 1.30–1.22 (m, 1H), 0.92 (t, J = 7.4 Hz, 3H) (Figure 1A). Crucially, the integration ratio between the sialic acid protons (δ 3.79–3.61) and the CPT methylene protons (δ 5.31) was approximately 1:1, indicating a high conjugation efficiency without significant byproduct formation. The molecular weight of SA-SS-CPT was 972.25, and it showed 971.25 in the negative mode, as shown in Figure 1B. Infrared spectroscopic analysis of SA-SS-CPT revealed characteristic functional group absorptions: a peak at 3440 cm^−1^ corresponding to the O-H stretching vibration of hydroxyl groups, 3298 cm^−1^ to the N-H stretching vibration of secondary amines, 2735 cm^−1^ to the C-H stretching vibration of methyl groups, 1749 cm^−1^ to the C=O stretching vibration of carbonyl groups, 1263 cm^−1^ to the C-N stretching vibration, and 976 cm^−1^ to the C=C-H bending vibration of alkenes, as shown in Figure 1C. Crucially, the absence of the characteristic S-H peak near 2570 cm^−1^ indicated that the thiol groups had been completely consumed during the disulfide bond formation, confirming successful conjugation between SA and CPT via the -SS- linker. The maximum UV absorption peaks of SA-SS-CPT were observed at 257 nm, 338 nm, and 370 nm. Among these, the peaks at 338 nm and 370 nm appeared as doublets, while only the peak at 257 nm was a singlet. The presence of these peaks confirmed that the conjugated CPT retained its original conjugated ring structure, essential for its topoisomerase I inhibitory activity. Therefore, in subsequent experiments, the wavelength of 257 nm was selected as the maximum absorption wavelength for SA-SS-CPT, as indicated in Figure 1D. Intermediate compounds were characterized as exhibited in Figures S1–S5.

### 3.2. Characteristics of SA-SS-CPT Nanowires

**Figure 2:**
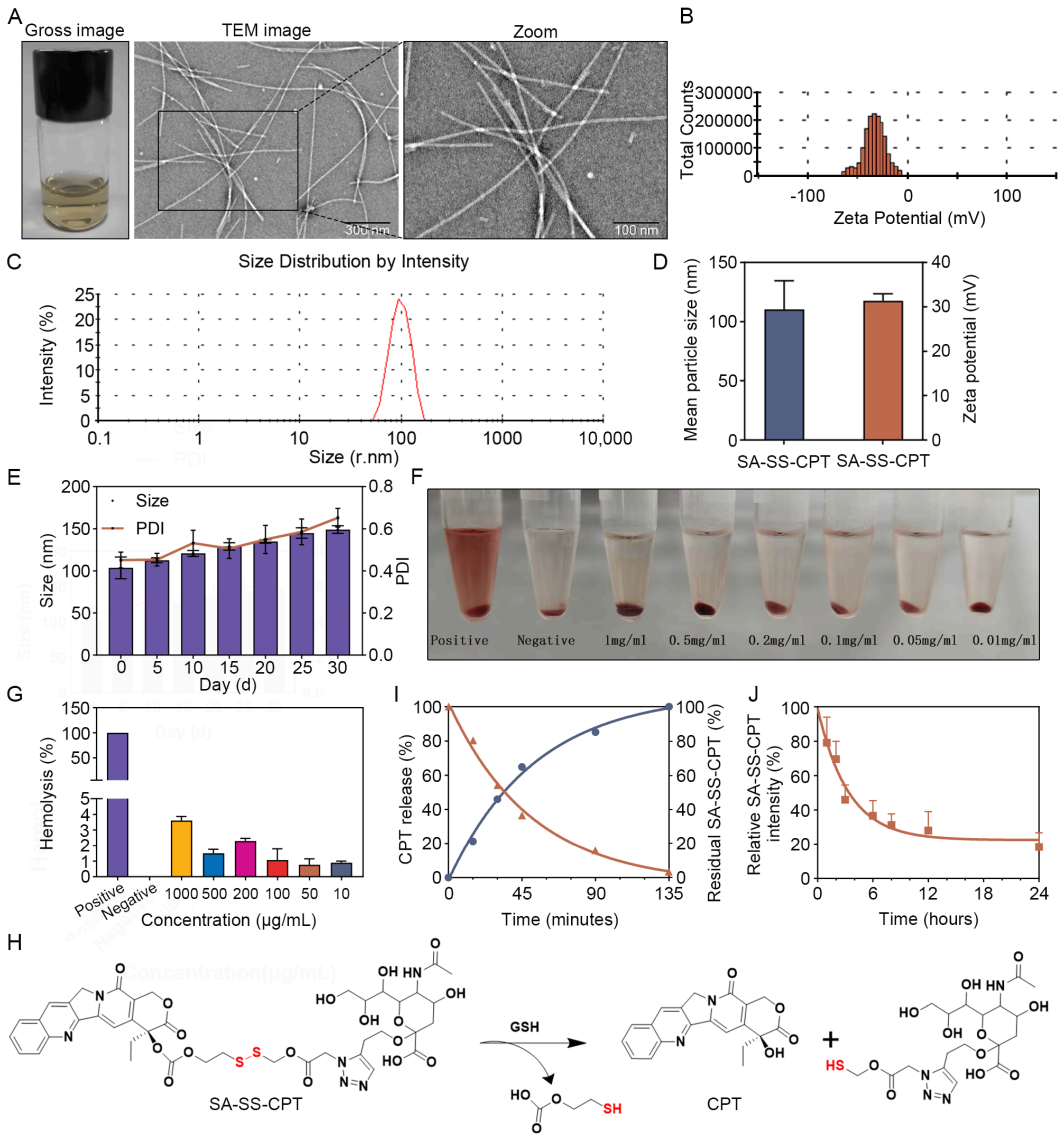
(**A**) Cross image and morphology of SA−SS−CPT nanowires observed at different magnifications. (**B**) Zeta potential (mV) of SA−SS−CPT nanowires. (**C**) Size distribution by intensity (%) of SA−SS−CPT nanowires. (**D**) Statistical analysis of the mean particle size and zeta potential of SA-SS-CPT nanowires. (**E**) 30-day stability of SA−SS−CPT nanowires. (**F**) Gross image of hemolysis. (**G**) Hemolysis (%) of SA−SS−CPT. (**H**) The mechanism of CPT release triggered by glutathione. (**I**) The CPT release profiles of SA−SS−CPT in the presence of GSH (10 μM), blue represents CPT release (%) and red represents residual SA−SS−CP. (**J**) The kinetics of SA−SS−CPT in the nude mice.

The aqueous solution of SA-SS-CPT nanowires appeared pale yellow. TEM observations revealed filamentous structures, presenting as either short fibers or continuous filaments with a diameter of approximately 10 nm, as shown in Figure 2A. Dynamic light scattering (DLS) analysis indicated that the SA-SS-CPT nanowires had an average hydrodynamic size of 110.4 ± 24.3 nm, a polydispersity index (PDI) of 0.259 ± 0.013, and an average zeta potential of −31.4 ± 1.6 mV, as shown in Figure 2B–D. Notably, the discrepancy between the TEM-derived diameter and the DLS-derived hydrodynamic size is expected and attributable to the fundamental differences between these two techniques. TEM measures the dehydrated, dry-state core diameter of individual nanowires, whereas DLS measures the hydrodynamic diameter of the hydrated nanostructures, including the contribution of the surrounding solvent layer. Moreover, DLS is sensitive to the formation of loose nanowire entanglements or aggregates in solution, which can significantly increase the apparent hydrodynamic size, while TEM captures only discrete, well-dispersed filaments following sample drying. The stability of the SA-SS-CPT nanowires was monitored over a storage period of 30 days. Although the particle size gradually increased with time, the overall size remained below 150 nm, which is consistent with nanoscale characteristics, as shown in Figure 2E. Hemolysis assays showed that the hemolysis rate of SA-SS-CPT at a concentration of 1000 μg/mL was significantly lower than 5%, indicating its safety for tail vein administration, as shown in Figure 2F,G. As demonstrated in Figure 2H,I, in vitro release studies of SA-SS-CPT revealed that upon treatment with 10 μM GSH, the disulfide bond was cleaved, leading to CPT release. The amount of released CPT gradually increased over time, reaching 100% at 135 min, while the residual SA-SS-CPT exhibited an opposite, decreasing trend. This rapid release kinetics is considered optimal for this application, as it ensures that upon internalization into TAMs, the therapeutic payload (CPT) is liberated quickly enough to induce effective DNA damage before being cleared by intracellular efflux pumps. Conversely, in the absence of GSH (PBS control), the nanowires showed minimal leakage (<5%), confirming excellent extracellular stability. Pharmacokinetic results in nude mice demonstrated that after administration of SA-SS-CPT, the relative SA-SS-CPT intensity in serum detected at different time points gradually decreased over time and stabilized after 12 h, as shown in Figure 2J.

### 3.3. Targeting Evaluation of SA-SS-CPT Nanowires

**Figure 3:**
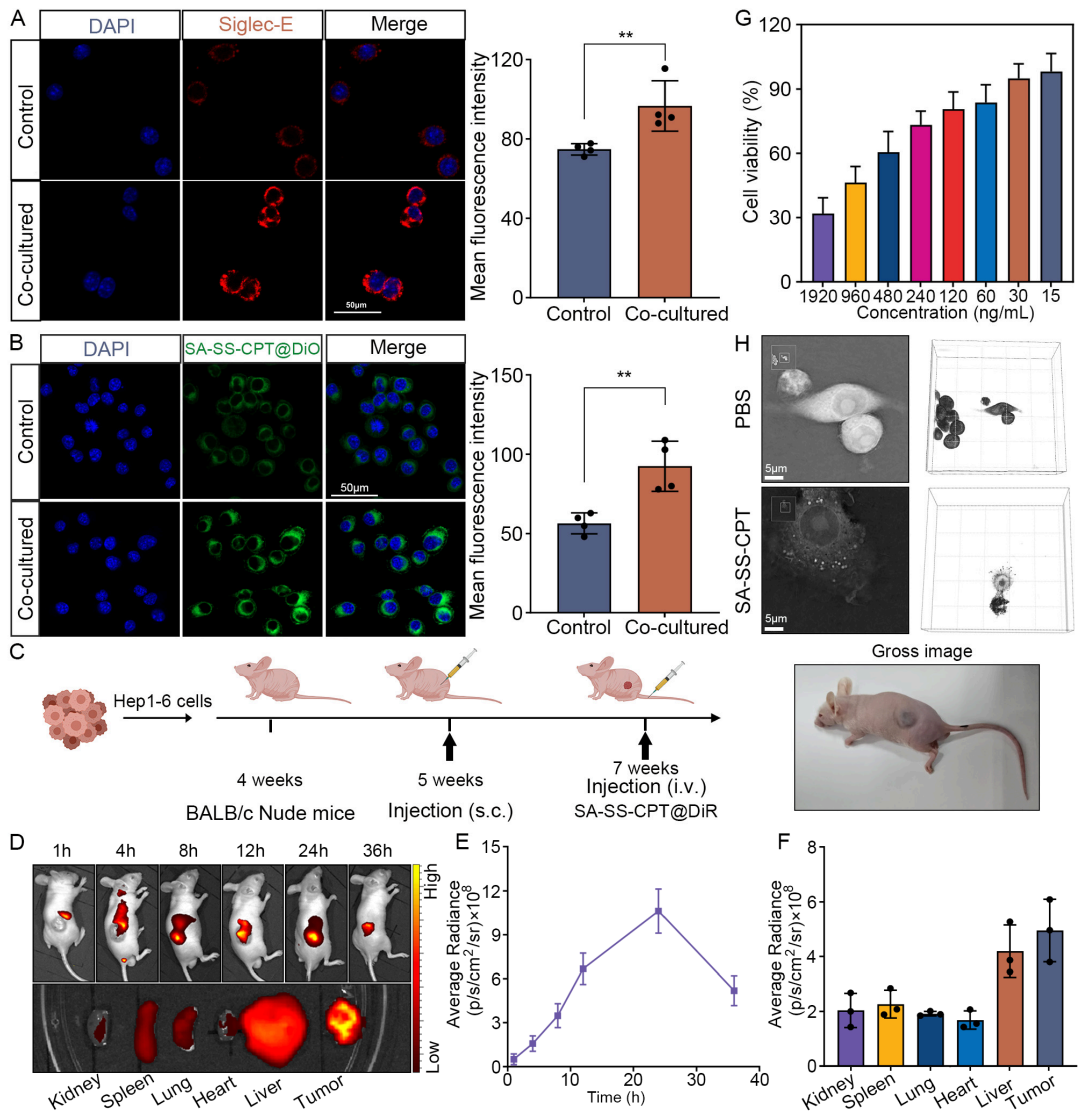
(**A**) Fluorescence expression and mean fluorescence intensity of Siglec-E, comparison between two groups, ** *p* < 0.01. (**B**) In vitro cell uptake and representative images of SA−SS−CPT @DiO, and mean fluorescence intensity, comparison between two groups, ** *p* < 0.01. (**C**) Schematic diagram of the subcutaneous tumor model in nude mice and SA−SS−CPT @DiR intervention. (**D**–**F**) In vivo fluorescence images and semiquantitative study of excised organs and tumors at 36 h post-injection. (**G**) Cell viability (%) of SA−SS−CPT nanowires. (**H**) 3D nanolive image of Macrophage morphology.

Siglec-E, a receptor that recognizes sialic acid groups, can regulate various immune responses. Studies have shown that the expression level of Siglec-E significantly increases during the differentiation of monocytes into macrophages [22]. After co-culturing Hepa1–6 cells with RAW264.7 cells, the fluorescence expression of Siglec-E in RAW264.7 cells was significantly enhanced, with a notable difference in fluorescence intensity, as shown in Figure 3A. Similarly, the protein expression of Siglec-E in RAW264.7 cells also increased after co-culture, as shown in Figure S6. The highly expressed Siglec-E in RAW264.7 cells can specifically recognize sialic acid-modified SA-SS-CPT nanowires. After encapsulating DiO dye with SA-SS-CPT nanowires (SA-SS-CPT@DiO), the nanowires were administered to both RAW264.7 cells and co-cultured RAW264.7 cells for 4 h. Confocal imaging revealed that the uptake of SA-SS-CPT@DiO was significantly higher in co-cultured RAW264.7 cells compared to the control group (Figure 3B).

To evaluate the in vivo tumor targeting ability of SA-SS-CPT, a subcutaneous tumor model was first established in nude mice. Hepa1–6 cells were suspended and injected subcutaneously into 5-week-old nude mice. After successful modeling, SA-SS-CPT encapsulating DiR (SA-SS-CPT@DiR) was administered via tail vein injection in the 7th week (Figure 3C). Initially, SA-SS-CPT@DiR primarily accumulated in the liver of the nude mice. Over time, after 8 h, SA-SS-CPT@DiR gradually accumulated at the tumor site, reaching its peak at 24 h. The distribution of SA-SS-CPT@DiR in various organs of the nude mice was examined, and it was observed that SA-SS-CPT@DiR mainly accumulated in the liver and tumor (Figure 3D–F).

To determine the optimal concentration of SA-SS-CPT for influencing RAW264.7 cells polarization while ensuring cell survival, RAW264.7 cells were incubated with SA-SS-CPT for 24 h, and cell viability was measured. The results showed that at a concentration of 1.95 μg/mL, the cell survival rate was 31.9 ± 7.3%. However, at a concentration of 30 μg/mL, the cell survival rate was 91.3 ± 9.8%, and at 15 μg/mL, it was 98.2 ± 8.3%, as shown in Figure 3G. Therefore, the optimal concentration of SA-SS-CPT for subsequent experiments was determined to be 30 μg/mL. In addition, we evaluated the toxicity of SA-SS-CPT to the heart, liver, spleen, lung and kidney of mice in vivo. The results showed that SA-SS-CPT was non-toxic to the heart, liver, spleen, lung and kidney of mice (Figure S7).

### 3.4. SA-SS-CTP Induces DNA Damage-Dependent M1 Macrophage Polarization

We employed 3D nanolive imaging to observe the polarization state of RAW264.7 cells 24 h after SA-SS-CPT intervention. Unpolarized RAW264.7 cells typically exhibited a regular spherical or elliptical morphology with a relatively smooth surface. They displayed fewer and shorter pseudopodia (cellular protrusions), and intracellular organelles such as lysosomes and mitochondria were evenly distributed without obvious polarity. However, after 24 h of SA-SS-CPT treatment, the RAW264.7 cells transitioned to a pro-inflammatory (M1) phenotype which generally showed an irregular, flattened morphology with increased spreading area, more filopodia and lamellipodia at the periphery, and distinct membrane ruffling structures in 3D imaging (Figure 3H).

**Figure 4:**
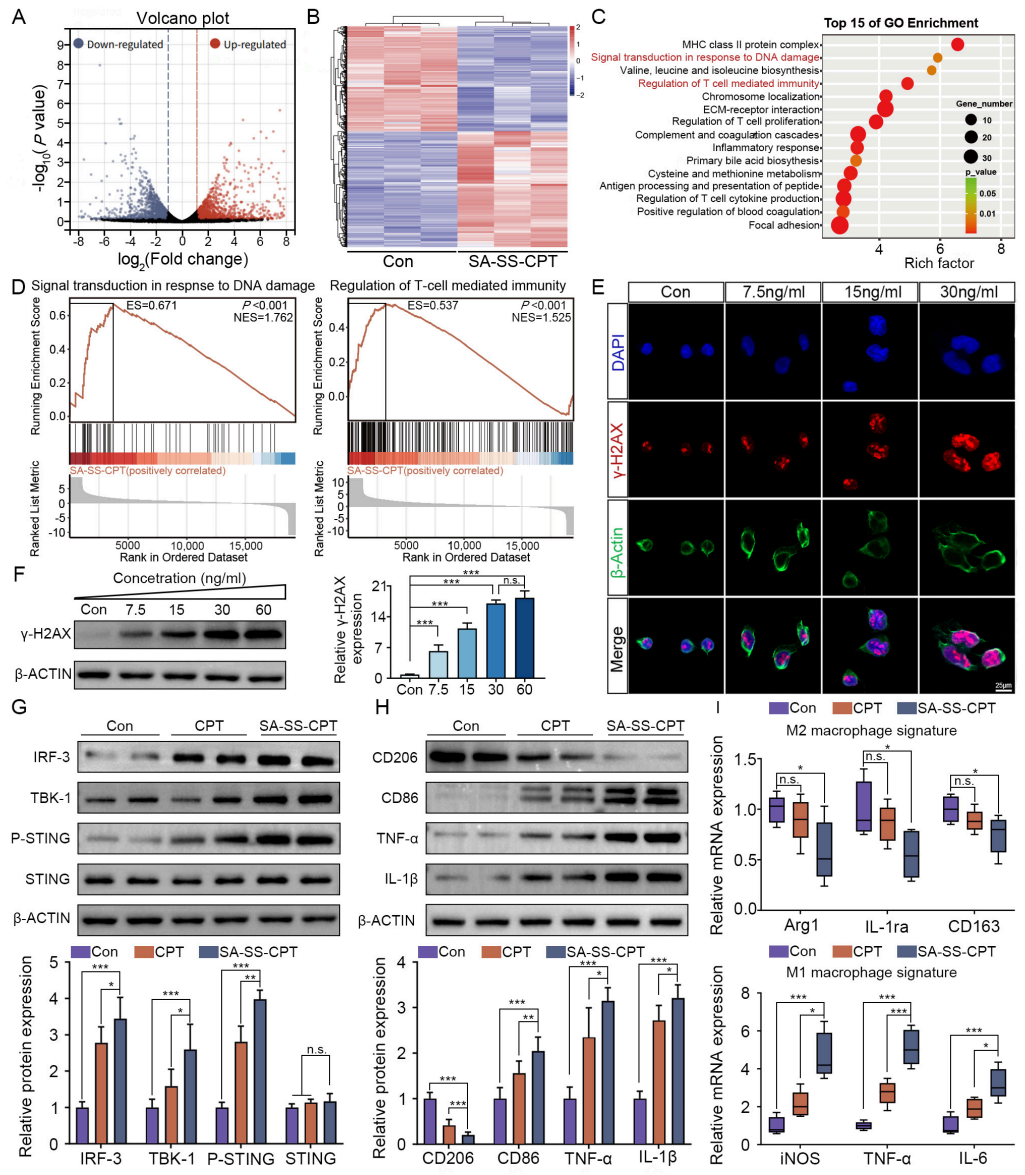
(**A**) Volcano plot of differential genes. (**B**) Heat map representing the significantly regulated genes detected in RNA-seq analysis of control and SA−SS−CPT and gene expressions were normalized with row Z-score. (**C**) Kyoto Encyclopedia of Genes and Genomes (KEGG) pathway enrichment analysis in macrophages compared with macrophages treated with SA−SS−CPT for 24 h. (**D**) Gene Set Enrichment Analysis (GSEA) of regulated genes in macrophages and the SA−SS−CPT group. Signal transduction in response to DNA damage, *p* < 0.001, ES = 0.671, NES = 1.762, and regulation of T-cell mediated immunity, *p* < 0.01, ES = 0.537, NES = 1.525. (**E**) Fluorescence imaging of γ-H2AX. (**F**) Western blot band images and relative gray-scale statistical graph of γ-H2AX protein expression, *** *p* < 0.001. (**G**) Western blot band images of IRF-3, TBK-1, P-STING, STING expression and relative protein gray-scale statistical graph in three groups, * *p* < 0.05, ** *p* < 0.01, *** *p* < 0.001. (**H**) Western blot band images of CD206, CD86, TNF-α, IL-1β expression and relative gray-scale statistical graph in three groups, * *p* < 0.05, ** *p* < 0.01, *** *p* < 0.001. (**I**) Relative mRNA expression of Arg-1, IL-1ra, CD163, iNOS, TNF-α, IL-6 in three groups, * *p* < 0.05, *** *p* < 0.001, n.s., not significant.

To investigate the mechanism by which SA-SS-CPT administration promoted polarization of RAW264.7 cells, RNA sequencing was carried out using total RNA extracted from RAW264.7 cells treated with SA-SS-CPT for 24 h. Impressive alternations in the gene expression profiles of RAW264.7 cells were detected (Figure 4A,B). Significantly different expressed genes were filtered and enriched in the GO signaling pathway. Notably, the top 15 signaling pathways were listed and we found that the enriched gene set was associated with signal transduction in response to DNA damage and T-cell immunoregulation (Figure 4C,D). As γ-H2AX is a key marker of DNA damage, we measured its expression in RAW264.7 cells under different concentrations. The IF showed that RAW264.7 cells treated with increasing concentrations of SA-SS-CPT resulted in a concentration-dependent rise in γ-H2AX expression (Figure 4E). Consistent results were also observed in WB (Figure 4F).

Although SA-SS-CPT induces DNA damage in RAW264.7 cells, the underlying pathway through which DNA damage influences polarization remained unclear. To investigate this, we further examined the status of the cGAS-STING pathway following DNA damage by measuring the expression of key proteins including IRF-3, TBK-1, P-STING, and STING. Compared to the control group, both the CPT and SA-SS-CPT groups showed significantly enhanced expression of IRF-3, TBK-1, and P-STING. Notably, the SA-SS-CPT group exhibited the strongest upregulation of these proteins relative to the CPT group, while STING expression remained consistent across all three groups. These results indicate that SA-SS-CPT treatment activates the cGAS-STING pathway in RAW264.7 cells, as shown in Figure 4G. After treatment with 30 μg/mL SA-SS-CPT, RAW264.7 cells displayed a shift toward M1 polarization, characterized by significantly reduced protein expression of CD206 and increased expression of the M1-related inflammatory cytokines TNF-α and IL-1β. Meanwhile, the M1-related marker CD86 was also elevated, with statistically significant differences among the groups, as shown in Figure 4H. To further evaluate polarization at the transcriptional level, RT-PCR was performed to measure marker gene expression in RAW264.7 cells treated with Con, CPT, or SA-SS-CPT. The results revealed that, compared to the normal and CPT groups, the SA-SS-CPT group showed significantly increased mRNA expression of M1 markers (iNOS, TNF-α, and IL-6) and decreased expression of M2 markers (Arg1, IL-1ra, and CD163) (Figure 4I). In summary, SA-SS-CPT triggers DNA damage in RAW264.7 cells, activating the cGAS-STING pathway and driving M1 polarization.

### 3.5. Tumor Proliferation Evaluation

**Figure 5:**
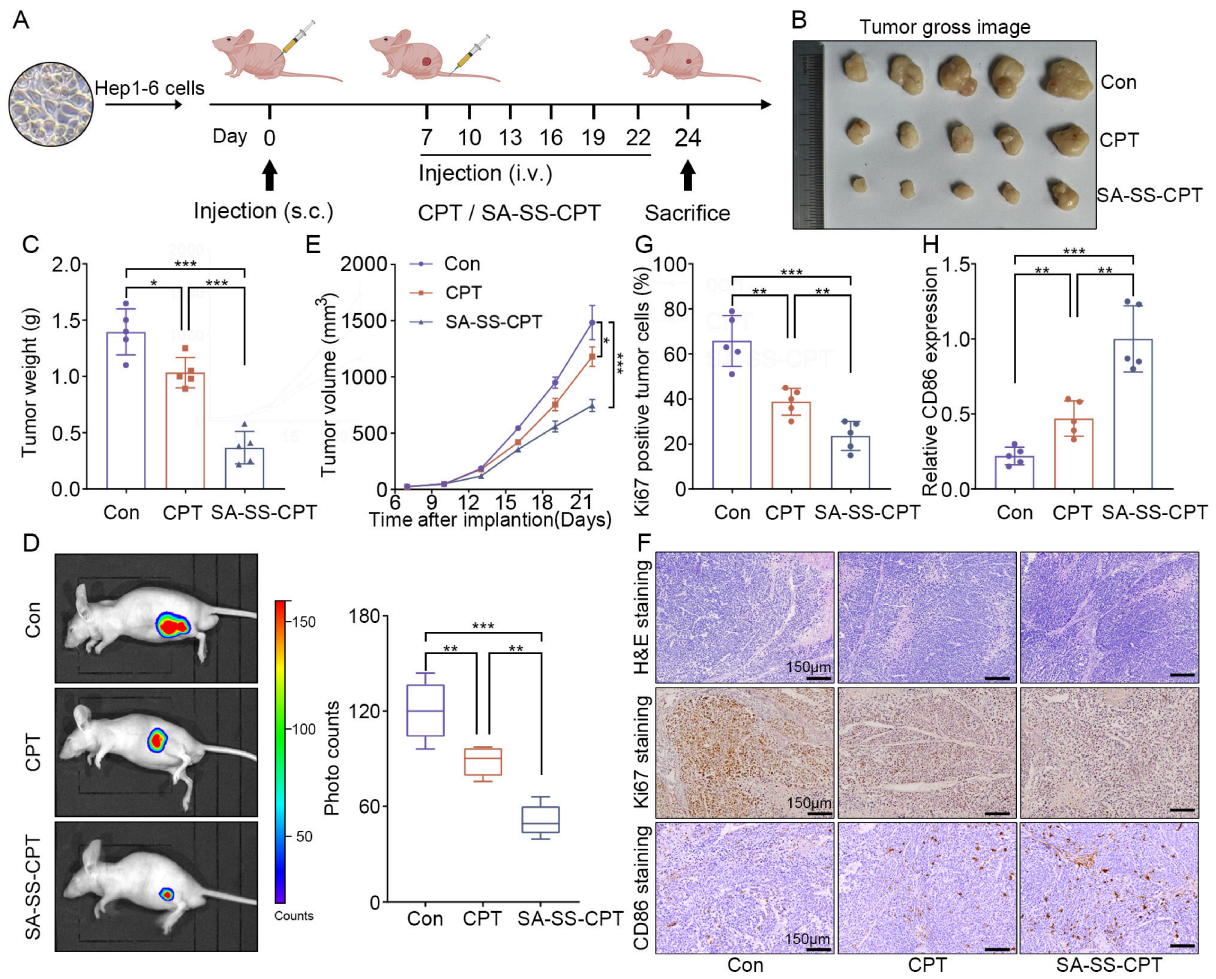
(**A**) Schematic illustration of the establishment of the animal model and treatment schedules. (**B**) Tumor gross image. (**C**) Tumor weight (g). (**D**) Tumor volume (mm^3^). (**E**) Tumor-bearing imaging. (**F**) HE staining, Ki67 staining, and CD86 staining; (**G**) Ki67 positive tumor cells (%). (**H**) Relative CD86 expression. Comparison between two groups, * *p* < 0.05, ** *p* < 0.01, *** *p* < 0.001. Data represent mean ± standard deviation (SD) from *n* independent experiments (*n* = 3).

Seven days after subcutaneous injection of Hepa1–6 cells into nude mice, the model was successfully established. The mice were then administered 1.0 μg/kg SA-SS-CPT via tail vein injection for 15 consecutive days, as shown in Figure 5A. Tumors were collected from each group and compared in size. The gross image of tumor tissues in the indicated group exhibited that SA-SS-CPT was the most effective in inhibiting tumor growth (Figure 5B). In addition, liver weight and bioluminescence imaging showed that the proliferation of tumor was slowed down significantly in SA-SS-CPT-treated mice compared to the mice model group and CPT group (Figure 5C,D). Similar results were observed that the tumor grew curve in mice treated with SA-SS-CPT were obviously fewer and smaller than that in model group and CPT group (Figure 5E).

Moreover, H&E staining and Ki67 staining indicated that tumor sections from model and CPT group mice presented stronger Ki67 staining than those from SA-SS-CPT group (Figure 5F,G). Furthermore, the M1-modulating role of SA-SS-CPT was further confirmed by IHC staining of CD86 (M1) at tumor tissues in the indicated groups (Figure 5F–H). CD86, as a co-stimulatory molecule, is primarily expressed on antigen-presenting cells such as macrophages and provides essential co-stimulatory signals for T cell activation and survival [23]. IHC staining of CD86 showed very low expression in the control group, with almost no observable reddish-brown spots. However, after intervention with CPT and SA-SS-CPT, the number of reddish-brown spots gradually increased, particularly in the SA-SS-CPT group. This suggested that SA-SS-CPT promoted the polarization state of macrophages.

### 3.6. Evaluation of Tumor Metastasis

**Figure 6:**
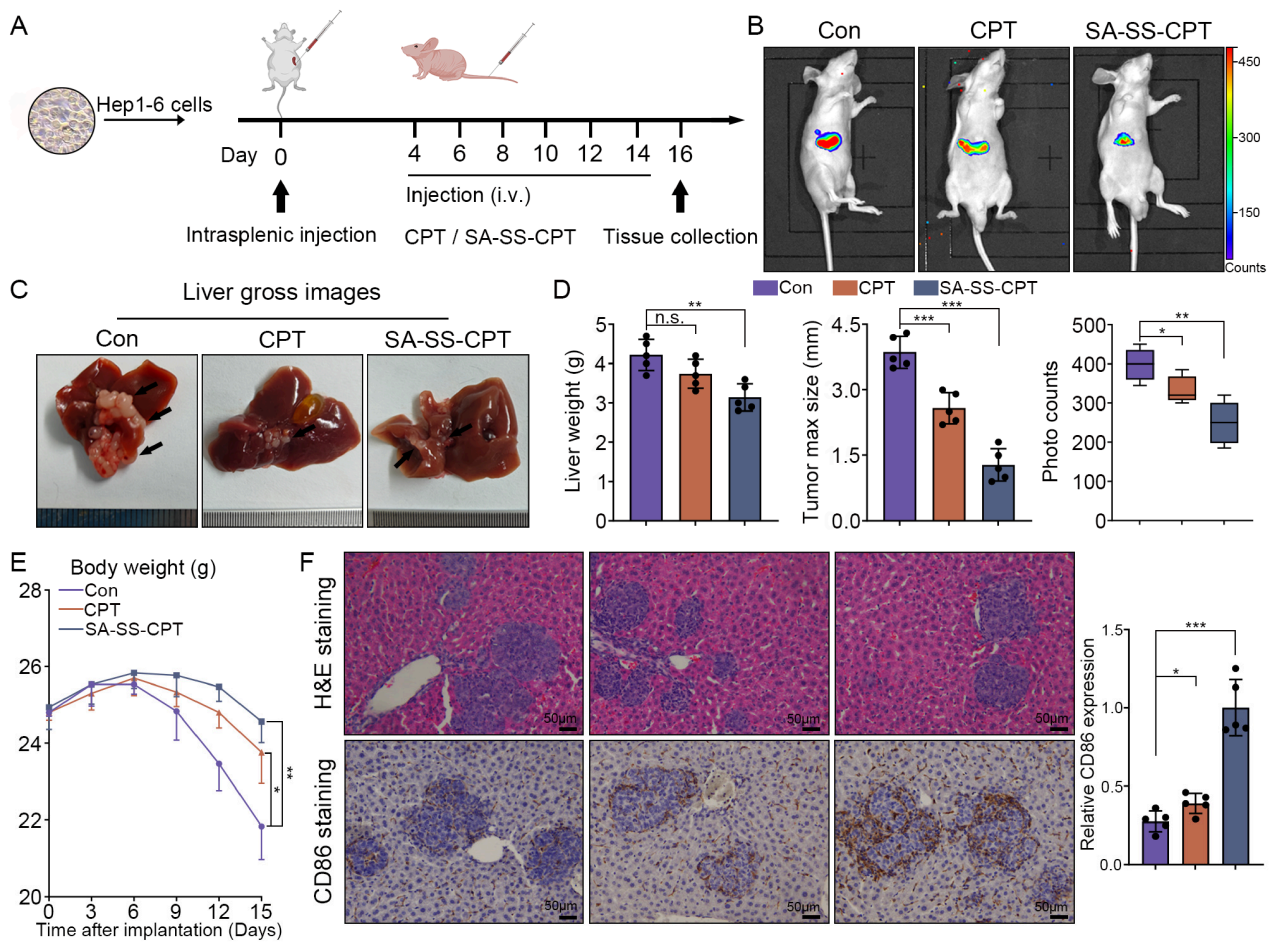
(**A**) Schematic showing the experimental design for the indicated group. (**B**) Tumor-bearing imaging. (**C**) Liver gross images. (**D**) Liver weight (g), tumor max size (mm), and photo counts. (**E**) Body weight (g). (**F**) H&E staining and CD86 staining, and relative CD86 expression. Comparison between two groups, * *p* < 0.05, ** *p* < 0.01, *** *p* < 0.001. Data represent mean ± standard deviation (SD) from *n* independent experiments (*n* = 3).

To further investigate the effect of SA-SS-CPT on tumor metastasis in vivo, we established a mouse liver metastasis model by intrasplenic injection of Hepa1–6 cells. Four days after the injection, interventions with CPT or SA-SS-CPT were administered via the tail vein every other day. Liver tissues were collected from each group on day 16, as shown in Figure 6A. Bioluminescence imaging revealed that the control group had the largest tumor burden with the highest bioluminescence intensity, followed by the CPT group. In contrast, the SA-SS-CPT group showed significantly reduced bioluminescence intensity and area (Figure 6B). The mice in the SA-SS-CPT group exhibited less and smaller liver metastatic nodules than the control group and CPT group (Figure 6C). Consistent results were observed that SA-SS-CPT treatment resulted in less liver metastatic burden in terms of liver weight and tumor maximum size (Figure 6D). We also monitored and analyzed the body weight changes of the mice across all groups. The results showed that from day 0 to day 3, the body weight of all groups exhibited an increasing trend. However, from day 6 to day 15, the body weight gradually decreased over time, with the less pronounced reduction observed in the SA-SS-CPT group (Figure 6E). The presence of the liver metastatic nodules was confirmed by H&E staining (Figure 6F). Furthermore, compared with the control group, CPT and SA-SS-CPT application resulted in stronger CD86 staining in liver tumor sections, particularly in the SA-SS-CPT group. Taken together, our findings indicated that SA-SS-CPT could suppressed HCC metastasis and promoted M1-macrophage infiltration in tumor tissues.

### 3.7. SA-SS-CPT Enhances the Immunotherapeutic Efficacy of PD-L1 in Mice

**Figure 7:**
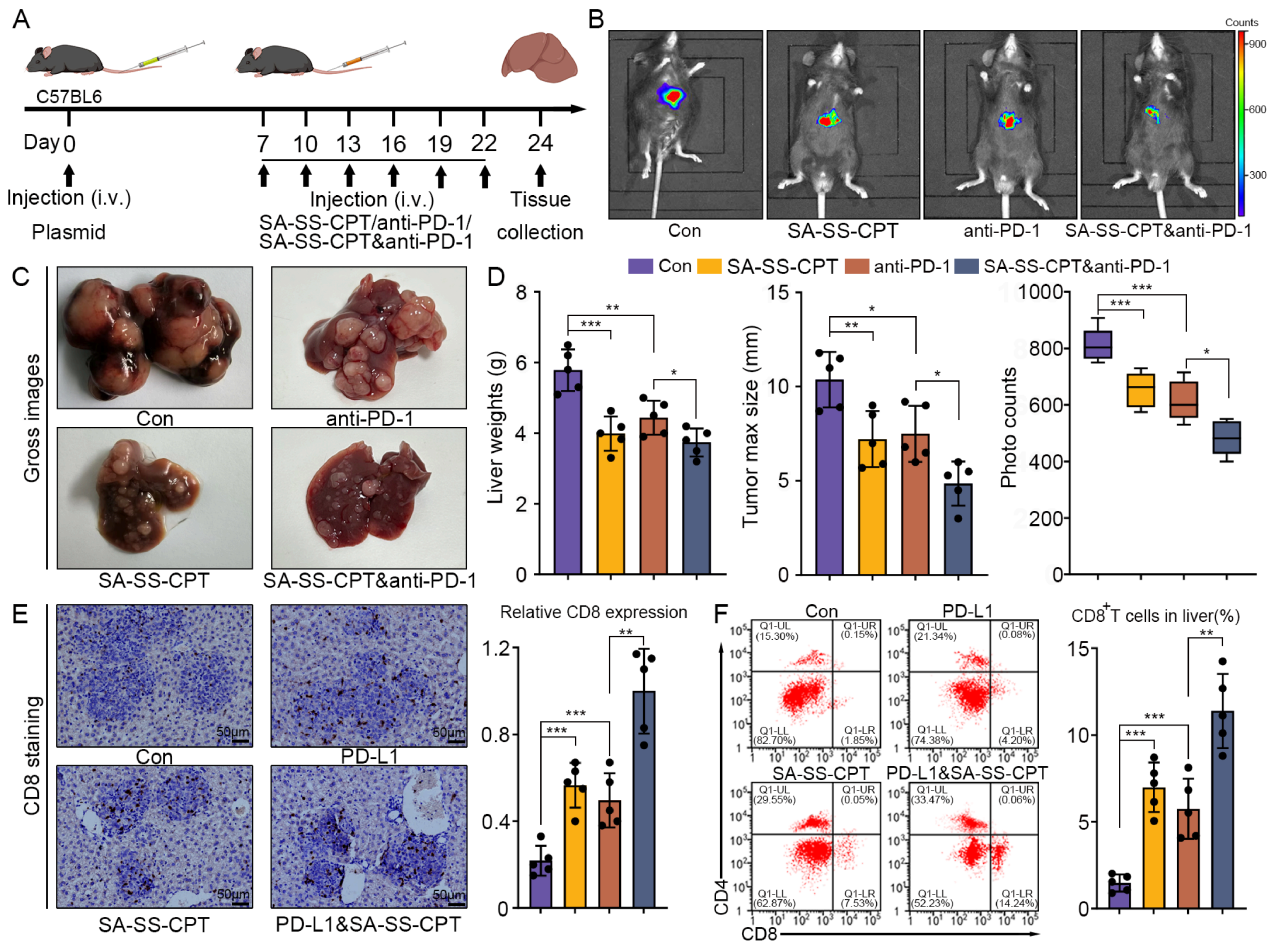
(**A**) The simplified experimental scheme for (**B**–**E**). (**B**) Tumor-bearing imaging. (**C**) Gross images. (**D**) Liver weights (g), tumor max size (mm) and photo counts, * *p* < 0.05, ** *p* < 0.01, *** *p* < 0.001. (**E**) CD8^+^ cells staining, ** *p* < 0.01, *** *p* < 0.001. (**F**) Flow cytometry and quantification of the portions of CD8^+^ and CD4^+^ population in macrophages, ** *p* < 0.01, *** *p* < 0.001.

The above observation provided evidence that SA-SS-CPT promoted the repolarization of TAMs from an M2 to an M1 phenotype. Hence, we investigated the therapeutic efficacy of combining SA-SS-CPT with anti-PD-1 therapy in mouse model of HCC. Following the successful establishment of hydrodynamic transfection HCC model, the mice were randomly assigned into four groups: control group, SA-SS-CPT group, anti-PD-1 group and SA-SS-CPT & anti-PD-1 group (Figure 7A). After treatment, the bioluminescence imaging showed that the combination therapy group mice suffered from the least tumor burden compared with mono-therapy groups (Figure 7B). Furthermore, we analyzed the number of HCC nodules on the liver surface as the evaluation standard, we found that the combination therapy group resulted in a lower liver tissue weight and a smaller maximum tumor nodule diameter in mice compared to the monotherapy groups, which were also reduced in the monotherapy groups relative to the control group (Figure 7C,D). We performed IHC staining on HCC tissues harvested from mice that received the respective treatments, and we found that compared to monotherapy groups, SA-SS-CPT & anti-PD-1 treatment resulted in an increased tumor infiltration of CD8^+^ T cells (Figure 7E). Consistently, CD8^+^ T cells in tumor tissues of mice treated with SA-SS-CPT & anti-PD-1 were approximately two times higher than those of mice received monotherapy (Figure 7F). Taken together, the data above indicated that SA-SS-CPT administration greatly improved the efficacy of checkpoint blockade therapy.

## 4. Discussion

Camptothecin is an important anticancer drug whose primary mechanism of action involves inhibiting the activity of topoisomerase I, thereby interfering with DNA replication and transcription and inducing tumor cell apoptosis. However, the clinical application of this compound is severely limited due to its poor water solubility and high toxicity. The low water solubility of camptothecin leads to insufficient bioavailability, which compromises its anticancer efficacy [24,25]. Furthermore, its active lactone ring is unstable under physiological conditions and prone to ring-opening reactions, resulting in drug deactivation [24]. To overcome these challenges, various nanocarrier systems have been developed to improve the solubility, stability, and controlled release behavior of camptothecin. For instance, cyclodextrin-based carriers can significantly enhance the solubility and stability of camptothecin through inclusion complexation [26]. Systems such as polymer microcapsules and nanomicelles contribute to improving its pharmacokinetic properties and targeting capabilities [27,28]. In recent years, smart responsive nanocarriers have garnered increasing attention due to their ability to trigger drug release under specific physiological conditions, thereby enhancing targeting and therapeutic efficacy [29,30]. Based on this, the present study designed an intelligent nanofilament system (SA-SS-CPT) responsive to GSH. These nanowires exhibit a filamentous morphology under transmission electron microscopy, with characteristics such as small particle size, high stability, and low hemolytic activity. This system not only effectively reduces the toxicity of camptothecin but also demonstrates good feasibility for in vivo applications.

SA-SS-CPT utilizes SA as a targeting ligand to achieve specific targeting of TAMs. Sialic acid can specifically bind to the Siglec-E receptor on the macrophage surface. This binding not only promotes high cellular uptake of the nanocarrier system but also competitively inhibits the immunosuppressive functions associated with M2 polarization, thereby reducing tumor immune evasion. The unique feature of the SA-SS-CPT nanowires designed in this study lies in their structural incorporation of sialic acid, which effectively targets Siglec-E and facilitates efficient internalization by macrophages via this pathway, preventing tumor immune evasion. Both confocal microscopy and flow cytometry results confirmed that SA-SS-CPT can specifically accumulate in TAMs. Without validation on primary human TAMs or in humanized mouse models, the translational potential of SA-SS-CPT remains speculative.

On the other hand, the disulfide bond (SS) structure in SA-SS-CPT confers GSH-responsive drug release capability. Under the high intracellular GSH concentration in TAMs, the disulfide bond undergoes reductive cleavage, leading to the release of CPT. In recent years, various GSH-responsive nanodrug delivery systems have been developed to achieve precise drug release in tumors and significantly enhance therapeutic efficacy [31,32,33,34]. However, the structure connecting sialic acid and camptothecin via a disulfide bond and self-assembling into nanowires has not been reported previously. In vitro experiments demonstrated that these nanowires can release CPT upon GSH triggering within TAMs. In vivo pharmacokinetic studies showed that the relative intensity of SA-SS-CPT in the serum of nude mice gradually decreased over time after administration and stabilized after 12 h. These results provide a basis for understanding the in vivo metabolic behavior of the drug and for developing rational dosing regimens.

To investigate the immune response mechanisms of macrophages following SA-SS-CPT intervention, transcriptome sequencing analysis revealed that differentially expressed genes in macrophages treated with SA-SS-CPT were enriched in pathways related to MHC class II protein complex, DNA damage, and T-cell immunoregulation. We observed an increase in γ-H2AX protein expression in macrophages after SA-SS-CPT treatment. As a well-established DNA damage marker, γ-H2AX is involved in the early recognition of DNA damage [35]. These results demonstrate that SA-SS-CPT significantly upregulates γ-H2AX levels in macrophages, induces DNA damage in TAMs, and consequently promotes their immunostimulatory functions.

In this study, SA-SS-CPT-induced DNA damage was found to activate the cGAS-STING pathway, as evidenced by increased expression of key pathway proteins including IRF-3, TBK-1, and phospho-STING. This indicates a close relationship between DNA damage and activation of the cGAS-STING pathway. As a DNA sensor, cGAS (cyclic GMP-AMP synthase) triggers innate immune responses by producing the second messenger cGAMP, which in turn binds to and activates STING (stimulator of interferon genes) [36]. Activation of the cGAS-STING pathway is not only associated with inflammatory responses but also closely linked to cellular senescence and cancer development [36]. Further analysis revealed that SA-SS-CPT treatment significantly upregulated both protein and gene expression of iNOS, TNF-α, and IL-6 in TAMs, indicating enhanced inflammatory responses. This increased inflammation subsequently contributed to further activation of the cGAS-STING pathway. Activation of this pathway plays a crucial role in remodeling the tumor microenvironment. As a key receptor for sensing both endogenous and exogenous DNA, the cGAS-STING pathway activates innate immunity and exerts important functions in antitumor immunity [37]. While multiple studies have shown that DNA damage can induce apoptosis via the cGAS-STING pathway [38], the SA-SS-CPT treatment dose used in this study (30 μg/mL) induced DNA damage in TAMs that was insufficient to trigger apoptosis, but instead promoted M1 polarization and initiated immune activation. Experimental results confirmed that activation of the cGAS-STING pathway following DNA damage in SA-SS-CPT-treated TAMs enhanced the expression of IRF-3, TBK-1, and phospho-STING, thereby amplifying inflammatory and antitumor immune responses and suppressing tumor immune evasion. These findings are consistent with recent studies highlighting the role of the cGAS-STING pathway in cancer immunotherapy [39,40].

To investigate the polarization of TAMs following SA-SS-CPT intervention, we examined TAM polarization markers and found that SA-SS-CPT promotes a shift toward M1 polarization. The interaction between M1-polarized macrophages and T cells plays a critical role in enhancing the efficacy of PD-L1 blockade therapy. This study demonstrates that the combination of PD-L1 blockade and SA-SS-CPT not only acts through the activation of CD8^+^ T cells but also mediates antitumor effects by remodeling the innate immune landscape within the tumor microenvironment, specifically by inducing M1-like macrophage polarization. These findings are consistent with previous studies confirming that promoting M1 polarization can potentiate the effectiveness of PD-L1 blockade therapy [41,42,43].

The antitumor efficacy of SA-SS-CPT was further validated in vivo. In a nude mouse tumor model, administration of 30 μg/mL SA-SS-CPT via tail vein injection for 14 days significantly reduced tumor weight and suppressed tumor cell proliferation, demonstrating the potent inhibitory effect of SA-SS-CPT on tumor growth. Furthermore, in the evaluation of tumor metastasis, SA-SS-CPT treatment markedly decreased the number of metastatic tumor cells in liver tissues. Concurrent analysis in the animal model confirmed that SA-SS-CPT promotes M1 polarization of macrophages.

In summary, this study successfully developed a multifunctional nanoplatform that intelligently integrates targeted drug delivery and immunomodulation. It not only achieves efficient tumor killing but also activates antitumor immune responses. These findings provide a novel strategy and experimental foundation for overcoming the clinical limitations of camptothecin and developing new combination immunotherapy approaches.

Despite these promising findings, several limitations of the present study should be acknowledged. First, while the SA-SS-CPT nanowires demonstrated effective targeting of TAMs via murine Siglec-E, this receptor is predominantly expressed in mouse macrophages. The corresponding functional orthologs in humans are Siglec-9 and Siglec-10. Although the sialic acid-binding domain is highly conserved among Siglec family members, suggesting that SA-SS-CPT may retain binding affinity for human Siglec-9, we acknowledge that differences in expression levels, ligand specificity, and downstream signaling between Siglec-E and its human counterparts may exist. Therefore, whether SA-SS-CPT exhibits comparable binding affinity and specificity for human Siglec-9 remains to be validated. Future studies employing primary human TAMs isolated from HCC patient specimens or humanized mouse models are warranted to assess the translational potential of this SA-mediated targeting strategy. Second, it is important to carefully delineate the conclusions that can be drawn from the nude mouse tumor models versus the immunocompetent PD-L1 blockade model. Since BALB/c nude mice are T-cell deficient, the tumor growth inhibition and increased M1-polarized macrophage infiltration observed in these experiments are primarily attributed to innate immune remodeling-specifically, the SA-SS-CPT-induced DNA damage in TAMs leading to their functional repolarization. These data demonstrate that SA-SS-CPT can modulate the innate immune compartment independently of adaptive immunity. However, the full spectrum of adaptive antitumor immunity, particularly the activation of cytotoxic CD8^+^ T cells and their contribution to long-term tumor control, cannot be assessed in the nude mouse model. Such conclusions are supported exclusively by the results obtained in the immunocompetent C57BL/6 mice receiving PD-L1 blockade, where enhanced CD8^+^ T-cell infiltration was observed.

## 5. Conclusions

In this study, we successfully developed a novel TAM-targeting nanoplatform, SA-SS-CPT, which self-assembles into GSH-responsive nanowires to overcome camptothecin’s poor solubility, bioavailability, and toxicity. By conjugating CPT to sialic acid via a disulfide bond, SA-SS-CPT achieves active TAM targeting through Siglec-E recognition and enables controlled CPT release upon internalization. The released CPT induces DNA damage in TAMs, activating the cGAS-STING pathway and driving functional repolarization of TAMs from the pro-tumoral M2 to the anti-tumoral M1 phenotype, thereby enhancing pro-inflammatory and anti-tumor immune responses while counteracting immune evasion. In vivo studies in liver cancer mouse models confirm that SA-SS-CPT significantly suppresses tumor growth and metastasis, promotes M1 macrophage infiltration, and synergizes with PD-L1 blockade to enhance CD8^+^ T cell-mediated tumor clearance. Collectively, this work establishes SA-SS-CPT as a promising immunostimulatory nanomedicine that repurposes CPT to remodel the tumor immune microenvironment, providing a robust foundation for TAM-targeted combination immunochemotherapy in hepatocellular carcinoma and potentially other solid tumors.

## Figures and Tables

**Table 1:** Gene-specific primers.

Name	Primers Sequences (5′------3′)
Arg-1	Forward: CAGCACTGAGGAAAGCTGGT
	Reverse: CAGACCGTGGGTTCTTCACA
IL-1ra	Forward: TAAGCAACCACCTTGAGCCT
	Reverse: CCAGCAGATTTCCATCCCGA
CD163	Forward: TGCTGTCACTAACGCTCCTG
	Reverse: CATTGCATGCCAGGTCATCG
iNOS	Forward: TGTCGCAGCTCCCTATCTTG
	Reverse: CATTGGCCAGCTGCTTTTGC
TNF-α	Forward: TGTCTCAGCCTCTTCTCATTCC
	Reverse: TGAGGGTCTGGGCCATAGAAC
IL-6	Forward: TAGTCCTTCCTACCCCAATTTCC
	Reverse: TTGGTCCTTAGCCACTCCTTC

## Data Availability

The datasets used and/or analyzed during the current study are available from the corresponding author upon reasonable request.

## Supplementary Materials

The following supporting information can be downloaded at: https://www.mdpi.com/article/10.3390/pharmaceutics18060649/s1, Scheme S1. The synthetic route of SA-SS-CPT. Figure S1. Liquid chromatography-mass spectrometry (LC-MS) of compound 2. Figure S2. LC-MS of compound 3. Figure S3. LC-MS of compound 4. Figure S4. LC-MS of compound 5. Figure S5. 1H NMR of compound 5. 1H NMR (400 MHz, MeOD) δ 3.96 (q, J = 9.0 Hz, 2H), 3.88 (d, J = 8.1 Hz, 1H), 3.81 (d, J = 11.5 Hz, 2H), 3.70 (d, J = 8.9 Hz, 2H), 3.50 (t, J = 7.6 Hz, 2H), 2.50 (d, J = 7.7 Hz, 2H), 2.40 (dd, J = 12.8, 4.2 Hz, 1H), 2.24 (t, J = 2.6 Hz, 1H), 2.03 (d, J = 14.8 Hz, 3H), 1.64 (t, J = 11.6 Hz, 1H). Figure S6. Western blot band images of Siglec-E expression and relative protein gray-scale statistical graph in two groups, ** *p* < 0.01. Figure S7. In vivo hepatorenal toxicity evaluation of SA-SS-CPT. Table S1. Pharmacokinetic parameters of SA-SS-CPT following a single tail vein injection in C57 mice (Mean ± SD, *n* = 3).
