# Supplementary material for: Camptothecin Nanowires Induce the cGAS-STING Pathway to Remold Tumor-Associated Macrophages for Antitumor Immunity
Source: Pharmaceutics. 2026 May 25;18(6):649. doi: 10.3390/pharmaceutics18060649 (PMC13304775; doi:10.3390/pharmaceutics18060649)
Supplement: Supplementary file 1 [file pharmaceutics-18-00649-s001.zip › pharmaceutics-4215948-supplementary.pdf]

Camptothecin nanowires induce the cGAS-STING pathway to remold tumor-associated macrophages for antitumor immunity

Congyi Zhang[1], Haotian Wu[2], Xiaotong Chen[3], Wenze Yin[2], Shizhuan Huang[2], Dixiang Wen[2], Xueting Song[3], Xiaoyan Xu[4], Changmei Zhang[4*], Sheng Tai[1*]

[1] Department of Hepatopancreatobiliary Surgery, Harbin Medical University Cancer Hospital, Harbin, China.
[2] Department of hepatic surgery, Second Affiliated Hospital of Harbin Medical University, Harbin, China.
[3] Department of Children's and Adolescent Health, Public Health College, Harbin Medical University, Harbin, 150086, China
[4] Department of Pharmaceutics, Harbin Medical University-Daqing Campus, 1 Xinyang Rd Daqing, 163319, China
* Correspondence: Changmei Zhang. Email: 710752984@qq.com；Sheng Tai. Email: taisheng1973@163.com

S1. Methods

S1.1 Synthesis of camptothecin nanowires

The synthetic route to Sialic Acid-SS-Camptothecin (SA-SS-CPT) is shown in Scheme S1. The structures of all intermediate compounds and the final product were confirmed by proton nuclear magnetic resonance (^{1}H-NMR, 400 MHz), and their purities were determined by MS-HPLC.

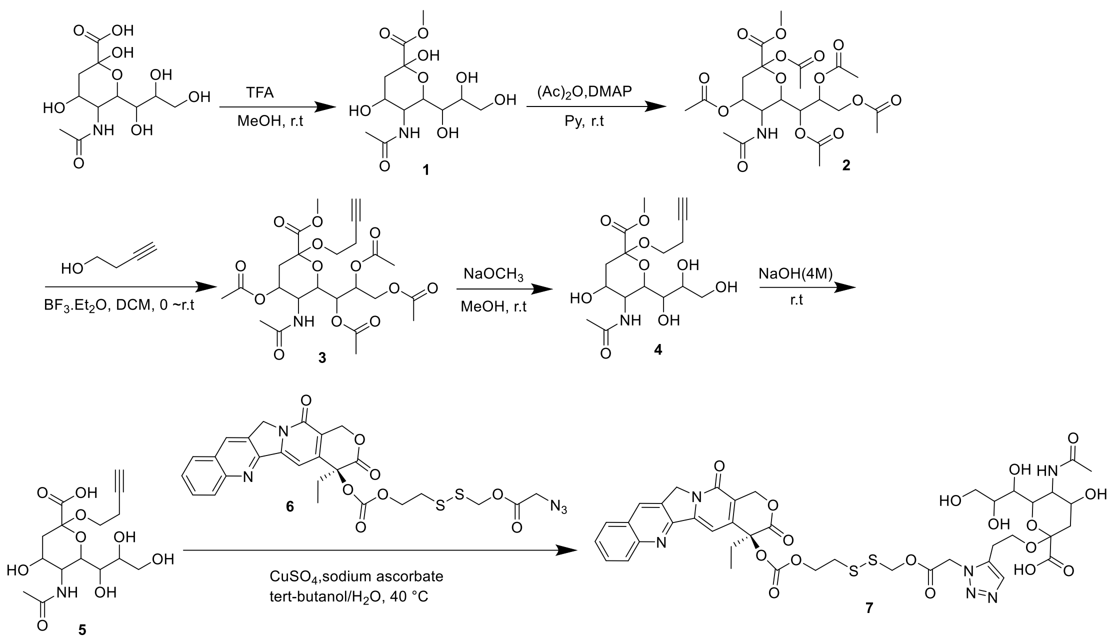

Scheme S1. The synthetic route of SA-SS-CPT.

S2. Results

S2.1 Characterization of intermediate compounds

The synthetic intermediates 2, 3, 4, and 5 were characterized and their structural identities confirmed by ^{1}H-NMR spectroscopy, as shown in Figures S1-S4. The ^{1}H-NMR spectrum of compound 5 is provided in Figure S5.

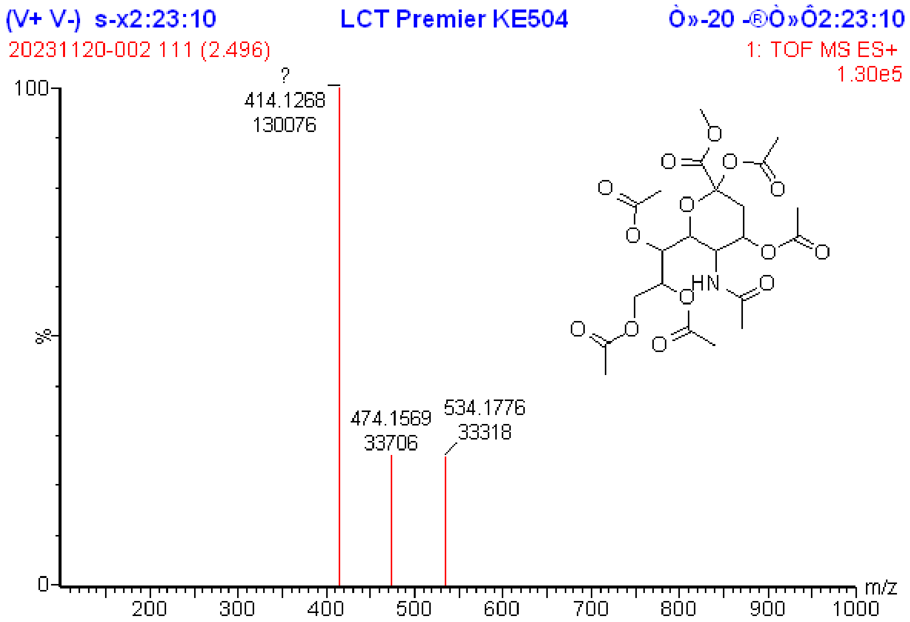

Figure S1. Liquid chromatography-mass spectrometry (LC-MS) of compound 2.

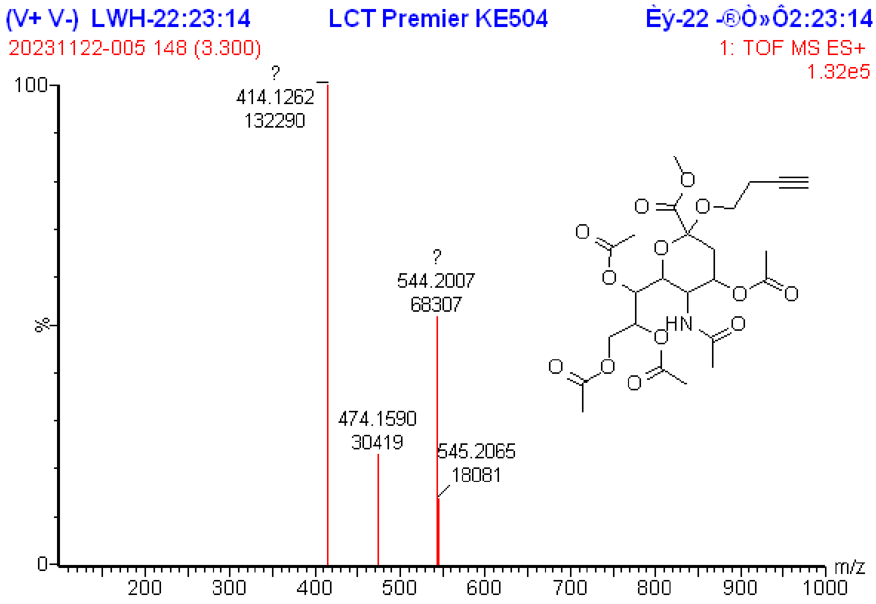

Figure S2. LC-MS of compound 3.

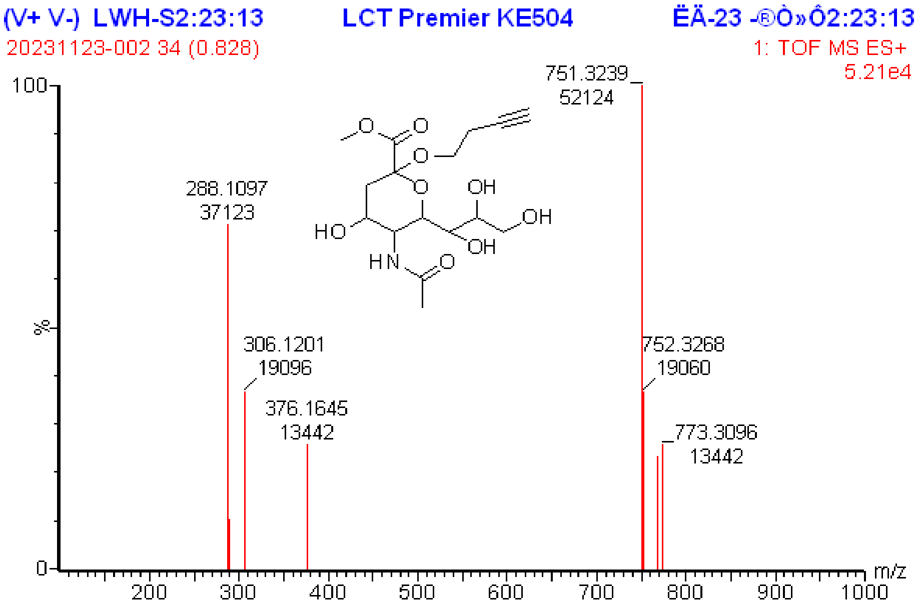

Figure S3. LC-MS of compound 4.

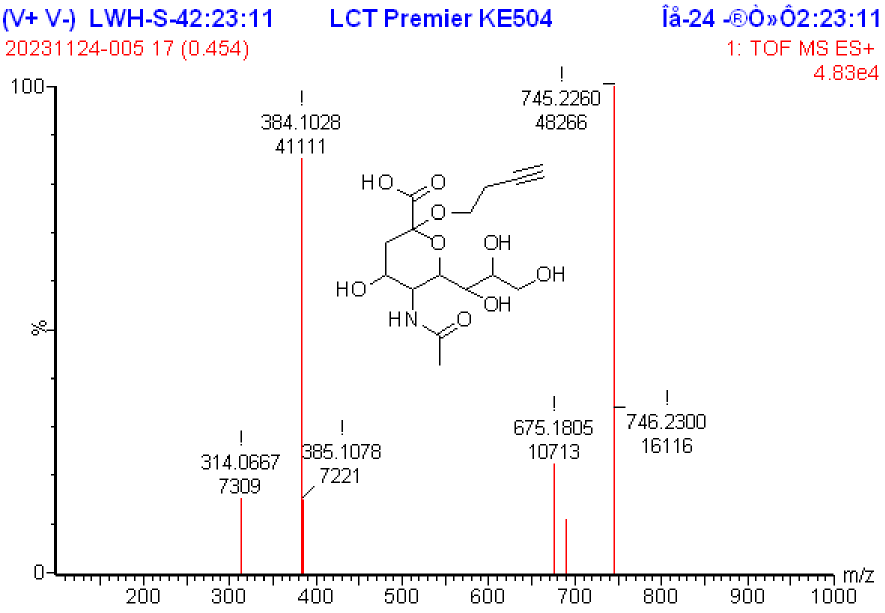

Figure S4. LC-MS of compound 5.

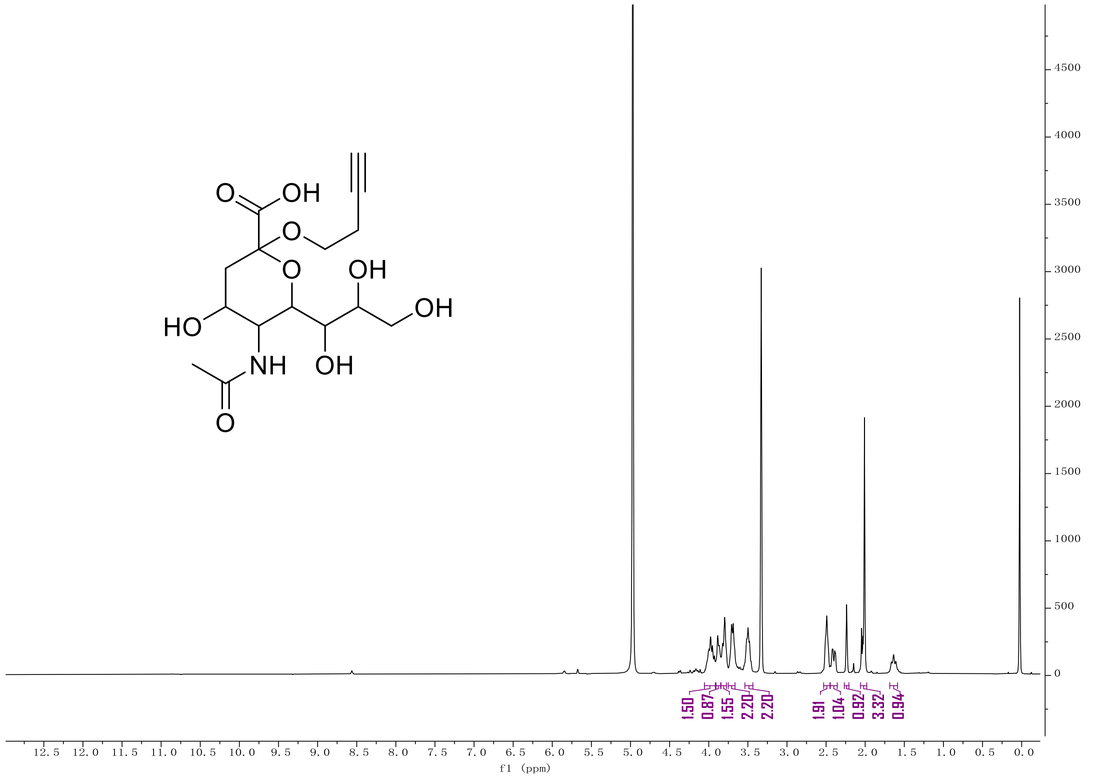

Figure S5. ^{1}H NMR of compound 5. ^{1}H NMR (400 MHz, MeOD) δ 3.96 (q, *J* = 9.0 Hz, 2H), 3.88 (d, *J* = 8.1 Hz, 1H), 3.81 (d, *J* = 11.5 Hz, 2H), 3.70 (d, *J* = 8.9 Hz, 2H), 3.50 (t, *J* = 7.6 Hz, 2H), 2.50 (d, *J* = 7.7 Hz, 2H), 2.40 (dd, *J* = 12.8, 4.2 Hz, 1H), 2.24 (t, *J* = 2.6 Hz, 1H), 2.03 (d, *J* = 14.8 Hz, 3H), 1.64 (t, *J* = 11.6 Hz, 1H).

S2.2 Expression of Siglec-E protein

Siglec-E protein expression was up-regulated in RAW 264.7 cells after co-culturing with hepa1-6 cells, compared with the control group of RAW 264.7 cells cultured in isolation, as shown in Figure S6.

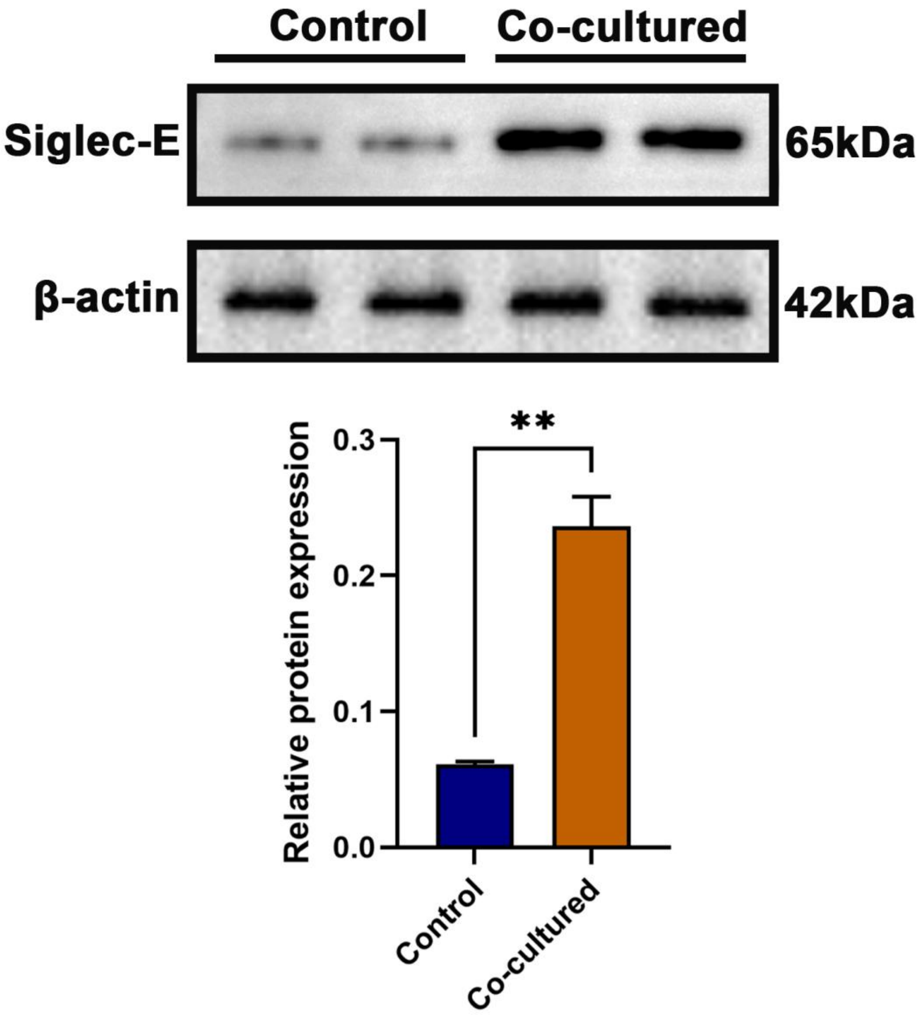

Figure S6. Western blot band images of Siglec-E expression and relative protein gray-scale statistical graph in two groups, $^{**}P<0.01$.

S2.2 Evaluation of hepatorenal toxicity by SA-SS-CPT

SA-SS-CPT was evaluated for potential hepatorenal toxicity by measuring the levels of alanine aminotransferase (ALT), aspartate aminotransferase (AST), blood urea nitrogen (BUN), creatinine (CR), creatine kinase (CK) and creatine kinase-MB (CK-MB). It was found that the conventional antitumor drug CPT exhibited potential hepatotoxicity, whereas SA-SS-CPT significantly alleviated this liver toxicity, as

shown in Figure S7A. Furthermore, H&E staining of the heart, liver, spleen, lungs, and kidneys of nude mice treated with SA-SS-CPT for 14 days revealed no pathological alterations in any of the observed organs, as presented in Figure S7B.

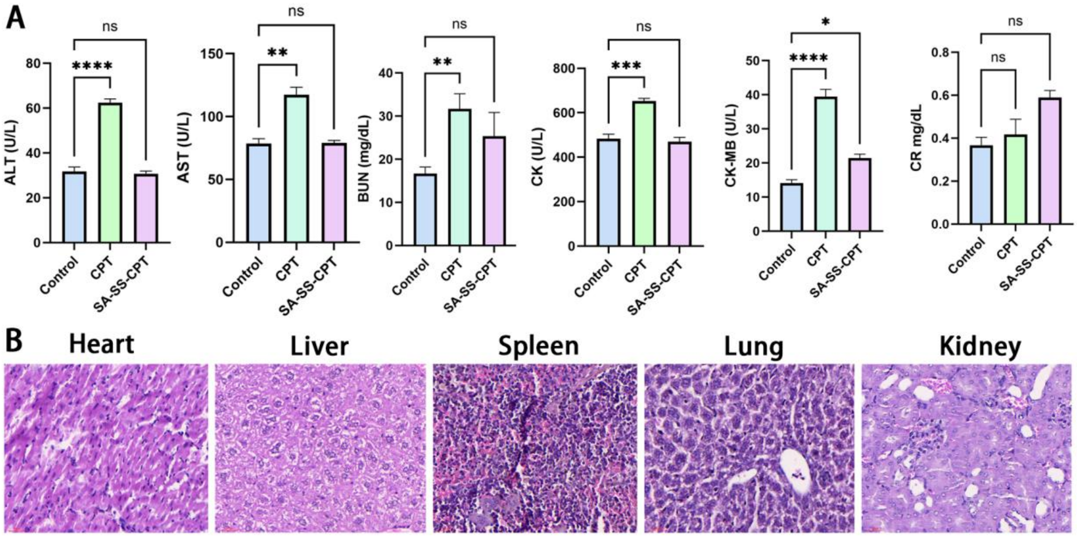

Figure S7. In vivo hepatorenal toxicity evaluation of SA-SS-CPT. (A) Measurement of serum ALT, AST, BUN, CR, CK, and CK-MB levels to assess the hepatorenal and cardiac toxicity; (B) Representative H&E-stained images of the heart, liver, spleen, lungs, and kidneys from nude mice following 14 days of treatment.

S2.3 Pharmacokinetic parameters of SA-SS-CPT

Table S1. Pharmacokinetic parameters of SA-SS-CPT following a single tail vein injection in C57 mice (Mean ± SD, n=3).

Time	AUC	Concentration(μg/mL)	Mean ± SD (μg/mL)	Half-life
0h	624.8, 605.2, 649.3	14.97, 14.48, 15.58	15.01 ± 0.55	30.0h
1h	472.3, 395.8, 539.2	11.18, 9.28, 12.84	11.10 ± 1.78	
2h	364.3, 421.9, 488.5	8.49, 9.93, 11.58	10.00 ± 1.55	
3h	299.1, 359.6, 251.3	6.87, 8.38, 5.68	6.98 ± 1.36	
6h	245.6, 311.8, 182.6	5.54, 7.19, 3.98	5.57 ± 1.61	
8h	212.3, 261.6, 171.4	4.72, 5.94, 3.70	4.79 ± 1.12	
12h	187.8, 258.2, 133.5	4.11, 5.86, 2.76	4.24 ± 1.55	
24h	123.9, 132.1, 256.2	2.52, 2.72, 5.81	3.68 ± 1.85	
